# Genomics of Caddisfly (Insecta: Trichoptera) Species Associated With Terrestrial Habitats

**DOI:** 10.1002/ece3.73327

**Published:** 2026-04-01

**Authors:** Jacqueline Heckenhauer, Charlotte Gerheim, Gabriela Jijon, Steffen U. Pauls, Robert W. Wisseman, Paul B. Frandsen

**Affiliations:** ^1^ Division of Terrestrial Zoology Senckenberg Research Institute and Natural History Museum Frankfurt Frankfurt Hesse Germany; ^2^ Senckenberg Research Institute and Natural History Museum Frankfurt Frankfurt Hesse Germany; ^3^ Department of Biology Brigham Young University Provo Utah USA; ^4^ Department of Plant and Wildlife Science Brigham Young University Provo Utah USA; ^5^ Institute of Insects Biotechnology Justus‐Liebig‐University Giessen Giessen Hesse Germany; ^6^ Aquatic Biology Associates, Inc. Corvallis Oregon USA

**Keywords:** evolution, genome assemblies, *h‐fibroin*, silk genes, transposable elements

## Abstract

Limnephilidae is a species‐rich and ecologically diverse family within the tube case making clade of caddisflies (Trichoptera). Species occur across a wide range of habitats, from cold headwater streams to stagnant pools to terrestrial environments. Here, we sequenced, assembled, and annotated whole genomes from two species that are associated with terrestrial environments: *Enoicyla pusilla* (Burmeister, 1839), distributed in the West Palearctic, and *Philocasca rivularis* (Wiggins, 1968), distributed in the Nearctic. Comprising three species, *Enoicyla* is the only genus of Trichoptera in Europe that exhibits a completely terrestrial life‐cycle. As one of four species of *Philocasca*, larvae of *P. rivularis* also exhibit terrestrial behavior during wet months, dispersing across the forest floor up to several meters away from the stream channel. In both species, we investigated genomic features, for example, genome size and dynamics of transposable elements across Limnephilidae. We also explored potential molecular adaptations of silk to terrestrial versus aquatic environments. Characterization of the major silk gene, *h‐fibroin*, of both species, as well as elemental analysis of silk of *Philocasca rivularis* , did not reveal molecular differences compared with silk of aquatic caddisfly species, potentially owing to the humid environments that both species inhabit. The new genomes form key resources for future genomic research on insect evolution, particularly related to habitat diversification and aquatic‐terrestrial transitions.

## Graphical abstract

Species of Limnephilidae (tube case making caddisflies (Trichoptera)) occur across a wide range of habitats, from cold headwater streams to stagnant pools to even terrestrial environments. We present high‐quality genome assemblies and annotations from two species that are associated with terrestrial environments: *Enoicyla pusilla* (Burmeister, 1839) and *Philocasca rivularis* (Wiggins, 1968). We used the newly generated genomes to investigate dynamics of transposable elements across Limnephilidae, determined the phylogenetic position of the two species, and explored potential molecular adaptations of silk to terrestrial versus aquatic environments. Our data form key resources for future genomic research in the field of insect evolution.

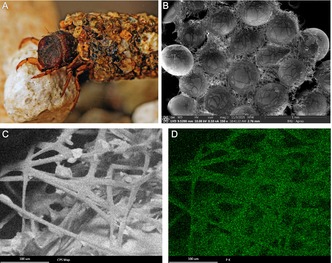

## 1 Introduction

Comprising more than 17,000 extant described species (Morse 2018) with ~50,000 species estimated (Schmid 1968), the primary aquatic insect order Trichoptera (caddisflies) represents one of the most species‐diverse freshwater animal radiations (Malm et al. 2013). Complementary to their species diversity, Trichoptera exhibit high ecological diversity owing to their microhabitat specialization, a full array of feeding modes and a diversity in silk‐spinning strategies (Dijkstra et al. 2014). Similar to their mainly terrestrial sister order Lepidoptera (moth and butterflies), caddisfly larvae produce silk from labial glands. The diversity of silk use generally aligns with the evolution of major caddisfly lineages, specifically at the subordinal level (Frandsen et al. 2024): Larva of suborder Annulipalpia create capture nets and stationary shelters of silk, often with attached mineral particles or plant material, which are fixed to the substrate (e.g., stones or aquatic plants). Some species of suborder Integripalpia (cocoon makers) build semipermeable pupal cocoons with an internal osmotic environment, while other species construct portable, tubular cases made purely from silk or from diverse materials encountered in their habitats, for example, sand grains, rock fragments, mollusk shells, or plant material, that are “glued” together by silk (tube case makers). Tube cases have been central to the diversification and evolutionary success of the order enabling camouflage and physical protection against predation during foraging. In addition, they enhance respiratory efficiency by channeling water through the case and across the abdominal gills (Wiggins 2004).

Limnephilidae is a particularly species‐rich and ecologically diverse family within the tube case making clade. Its species richness is reflective of both broad and highly specialized ecological adaptations. Across the family, species occur in a wide range of lotic, lentic, and temporary freshwater habitats, from turbulent perennial streams high in oxygen, to lentic environments low in oxygen, to temporary pools and streams, or even moist terrestrial environments. Some species are generalists, while others are highly specialized to narrow, and partly extreme aquatic habitats (e.g., Graf et al. 2008, 2025; Kuemmerlen et al. 2025). Here, we sequenced, assembled and annotated de novo genomes from two Limnephilidae species that are associated with extreme conditions along this gradient, that is, terrestrial environments: *Enoicyla pusilla* Burmeister, 1839 and *Philocasca rivularis* Wiggins, 1968 (in Wiggins and Anderson 1968).

The genus *Enoicyla* (subfamily Limnephilinae) comprises three described species ( *E. costae* McLachlan, 1876, *E. pusilla* and *E. reichenbachii* Kolenati, 1848) and is distributed in the West Palearctic region. *Enoicyla* is the only genus of Trichoptera in Europe that exhibits a completely terrestrial life‐cycle. *E. pusilla* larvae typically occur on deciduous forest floors, usually inhabiting the litter layer (Harding 1998), but has been also reported in plantations of native and non‐native pines (Lombardero 2013). Their habitat is typically concentrated around tree trunks, and larvae reportedly rarely venture more than 1 m from the tree where they were born (Harding 1998). Larvae have been observed to feed on relatively dry, undecomposed tree leaves, and occasionally on mosses and algae (Harding 1998).

The genus *Philocasca* (subfamily Philocascinae) consists of four described species, all confined to the Pacific Northwest of the USA: *P. banksi* Denning, 1941 from northern Idaho and northwest Montana; *P. demita* Ross, 1941, widely distributed in the Oregon Coast Range and in northern California coastal ranges, south to Humboldt County; *P. oron* Ross, 1949 known only from the type locality in the NW Oregon Coast Range; and *P. rivularis* widely distributed in the Oregon Coast Range, western Oregon Cascade Mountains, and in coastal ranges of northern California, south to Humboldt County. All four *Philocasca* species occur in or adjacent to first order, perennial, cool or cold, spring‐fed streams that are heavily shaded with a mixed conifer/hardwood overstory. They are found in ecoregions characterized by cool, wet winters with precipitation occurring mainly as rain from October/November to May/June. Summers are typically very dry. Anderson (1967) first reported the terrestrial behavior of *Philocasa demita* larvae. He found them to be entirely terrestrial, collecting them from forest litter or with pit‐fall traps up to 6 m from a small spring stream. Wiggins and Anderson (1968) reported larvae of *P. rivularis* in small rapid spring streams. Subsequently Wisseman (unpublished) frequently collected *P. rivularis* completely out of the stream. For this study, we kept larvae alive for weeks in a moist environment alone. Larval instars 1 to 5 disperse across the forest floor up to several meters away from the stream channel during wet months. During the dry summer, fifth instar larvae return to small perennial stream channels, where they spend the daytime resting beneath rocks and crawl onto the stream margins at night (Wisseman unpublished).

Both *Enoicyla pusilla* and *Philocasca rivularis* lay eggs terrestrially in gelatinous masses. A humid environment and adequate moisture are essential for successful egg development and the eclosion of first instar larvae from both the egg and the gelatinous matrix the eggs are embedded in. Desiccation can cause larvae to become trapped in the drying, sticky egg mass jelly and subsequently die (Harding 1998; Kelner‐Pillault 1960). For *P. rivularis* , fall rains are required to help liquify the mass matrix and activate larval emergence from the egg mass jelly (Wisseman unpublished).

Larval adaptation to terrestrial life is manifested in both morphology and behavior. For example, *Enoicyla* lacks tracheal gills and ion‐absorbing chloride epithelia typically found on the abdomen of limnephilid larvae (Wichard 1988). Respiration is enabled through the body surface, whose epidermis exhibits highly differentiated tracheation (Rathjen 1939). This type of respiration might explain humidity requirements. Experimentally, it has been shown that under uniform lighting, as well as in the dark, the larvae always crawl into the air layers with most moisture (up to 100% humidity). *Enoicyla* larvae also exhibit strong phototactic behavior, crawling away from the light when exposed to one‐sided illumination in the lab. In the wild, larvae live in moist leaf litter during the day. At night, they climb upwards onto tree trunks during humid weather, that is, after rain, before returning to leaf litter in the morning (Rathjen 1939). Larvae retreat back into the damp leaf litter when relative humidity drops below 70% (Kelner‐Pillault 1960). While Rathjen (1939) found final‐instar larvae to thrive best under lab conditions with a relative humidity of 100% (stenohydric), Harding (1998) demonstrated that earlier instars can survive for up to 3 weeks at humidities as low as 60%. In *Philocasca rivularis* , the larva's dorsally flattened and strongly sclerotized head functions like a snail's operculum when it is retracted into the case. While this serves as a defense mechanism against aquatic and terrestrial invertebrate predators, it might also be an adaptation to inhibit desiccation when the larva is terrestrial. Differing from *E. pusilla* , larvae of *P. rivularis* have single gills likely of use even when terrestrial when in/on damp substrates.

There is currently no genetic data publicly available for the genus *Philocasca*, and only short fragments of a few genes (COI, wingless, 18S) are available for *E. pusilla* (Gombeer et al. 2011; Morinière et al. 2017; Zhou et al. 2016). Thus, the high‐quality genomes presented here are key resources for future genomic research on Limnephilidae evolution and habitat diversification. In the present study, we used the newly generated genomes to investigate transposable elements (TEs), mobile genetic elements that can copy and transpose themselves into new genomic locations. While TEs are traditionally considered deleterious to their host's fitness (e.g., Mackay 1989), they play a role in the evolution of gene regulation (as reviewed in Feschotte 2008; Chuong et al. 2017) and in phenotype evolution and adaptation (see review Tossolini et al. 2025). Previous studies found expanded genomes with high amounts of TEs in caddisfly clades with higher ecological diversity (Olsen et al. 2021, Heckenhauer et al. 2023). Thus, TEs might form a genomic basis for habitat diversification in the ecologically diverse caddisfly family Limnephilidae.

Moreover, we examine the phylogenetic relatedness of *Enoicyla* and *Philocasca* within Limnephilidae. In addition, we explore molecular adaptations of caddisfly silk to terrestrial environments. Silk of aquatic caddisfly species has been previously studied (e.g., Stewart and Wang 2010). Full‐length sequences of the major silk gene, *h‐fibroin*, have been recently sequenced for 14 caddisfly species (Heckenhauer et al. 2023; Frandsen et al. 2023; Deng et al. 2025). Previous studies identified potential molecular adaptations of the h‐fibroin across Trichoptera that may be related to underwater silk use. These include ß‐sheet formation through bonding of a phosphorylated, repeating serine motif via complexation with metal cations obtained from the aquatic environment (Stewart and Wang 2010; Standring et al. 2025) and a high amount of charged amino acids potentially leading to less hydrophobicity (Heckenhauer et al. 2024). So far, the h‐fibroin of terrestrial caddisflies has not been studied. To close this knowledge gap, we identified the h‐fibroin of the two species occurring in terrestrial environments. We compared its primary structure and amino acid composition to the h‐fibroin of aquatic species. Moreover, we used elemental analysis to determine whether the silk of *P. rivularis* exhibits phosphorylation.

## 2 Materials and Methods

### 2.1 Sampling, DNA Extraction and Whole‐Genome Sequencing

An adult male individual of *E. pusilla* was collected into DNA/RNA Shield (Zymo Research) in Staatswald Bulau, Damburger Lache in Hesse, Germany (50.144410, 8.986116, permit number: V 53.2‐88 n 58/1432‐2020/3). High molecular weight genomic DNA was extracted from head and thorax tissue according to the protocol of Sambrook and Russell (2006). DNA concentration and DNA fragment length were assessed using the QuantiFluor ONE dsDNA System on the Quantus Fluorometer (Promega Corporation) and the Genomic DNA ScreenTape on the Agilent 4150 TapeStation system (Agilent Technologies), respectively. A library was prepared with the SMRTbell Express prep kit 3.0 (PacBio, Menlo Park, CA, USA) according to the instructions of “Preparing whole genome and metagenome libraries using SMRTbell prep kit 3.0” (PacBio, Version 01, April 2022). Total input DNA was approximately 820 ng. Quality control of the final library was again performed on the Quantus Fluorometer and the Agilent 4150 TapeStation system. Annealing of sequencing primers, binding of sequencing polymerase, and purification of polymerase‐bound SMRTbell complexes were performed using the Revio polymerase kit (PacBio, Menlo Park, CA, USA). The loading concentration for sequencing was 260 pM. Long‐read whole‐genome sequencing was performed on a PacBio Revio instrument (PacBio, Menlo Park, CA, USA), 24 h HiFi sequencing.

Larvae of *P. rivularis* were collected alive in southwest Oregon (42.72556, −124.39572) on 13 May 2023. Samples were transported to Brigham Young University in Provo, Utah where some were flash frozen with liquid N2. High molecular weight (HMW) DNA was then extracted from a single larval thorax using a Qiagen Genomic Tip Extraction kit. DNA was evaluated for quality using a Qubit fluorometer and for length on an Agilent FemtoPulse. HMW DNA was then sheared to 18 kbp with a Diagenode Megaruptor. A PacBio HiFi library was prepared using the SMRTbell prep kit 3.0. The library was then sequenced on a single SMRT cell on the Revio instrument at the BYU DNA Sequencing Center.

Software tools and commands used to perform the following analyses are given in Table S1.

### 2.2 Genome‐Size Estimation

We used a *k‐mer*‐based statistical approach for genome size estimation. After counting k‐mers with Jellyfish 2.3.0 (Marçais and Kingsford 2011) using *count* ‐C ‐s 1000000000 and a *k‐mer* length of 21 (−m 21) with the ccs‐reads, we generated a histogram of *k‐mer* frequencies, with *histo*. For genome profiling, that is, to estimate major genome characteristics such as genome size, heterozygosity, and repetitiveness, we used GenomeScope 2.0.0 (Ranallo‐Benavidez et al. 2020) with the *k‐mer* count histogram using the following parameters: *k‐mer* length (−k) = 21 and ploidy (−p) = 2.

### 2.3 Genome Assembly and Contamination Filtering

After we converted ccs reads from bam to fastq format using samtools 1.19.1 (Danecek et al. 2021) *bam2fq*, raw read statistics were calculated with fast_stats.py (https://github.com/sandyjmacdonald/fast_stats, last accessed 2025‐12‐10). We assembled ccs reads into contigs using hifiasm 0.19.5 ( *Philocasca rivularis* ) and 0.19.8 (Cheng et al. 2021, *Enoycila pusilla*) which generates a primary and an alternate assembly. Resulting gfa files were converted to fasta files with awk. We ran MitoHiFi 3.2 (Uliano‐Silva et al. 2023) on the primary assembly using the complete mitogenome of *Limnephilus decipiens* Kolenati, 1848 accession NC_026219.1 and excluded mitochondrial contigs detected by MitoHiFi. To screen for and filter out adaptor sequences in the nuclear genomes, we used run_fcsadaptor.sh of NCBI Foreign Contamination Screen (FCS, Astashyn et al. 2024) with parameter ‐‐euk.

In addition, we screened the nuclear genomes for potential contaminant sequences with FCS 0.5.5 using FCS‐GX *screen genome* the FCS‐GX database and ncbi tax‐ids “1271740” ( *E. pusilla* ) and “1683747” ( *P. rivularis* ). We filtered out contaminant sequences using FCS module *clean genome*. We used BlobToolkit 5.0.2 (Challis et al. 2020) to visualize the primary genome assemblies.

### 2.4 Assembly Quality Assessment

We evaluated contiguity of the final assemblies using QUAST 5.2.0 (Mikheenko et al. 2018) and completeness with compleasm 0.2.7 (Huang and Li 2023) using the lineage dataset endopterygota (odb12). We downloaded all available Limnephilidae genome assemblies from NCBI. Their contiguity and completeness are presented in Table 1.

**TABLE 1:** Quality metrics of available Limnephilidae genome assemblies.

Species (subfamily—tribe)	Accession number	Assembly length (Gb)	Contig/scaffold N50 (Mb)	No. of contigs/scaffolds	Compleasm, S: single; D: duplicated; F: fragmented; M: missing; *N*: 3754
*Eubasilissa regina* McLachlan, 1871 (outgroup, family: Phryganeidae)	GCA_022840565.1 (Kawahara et al. 2022)	0.92	32.4/n.a.	123/n.a.	S: 97.47% D: 0.32% F: 0.99% M: 1.23%
*Drusus annulatus* Stephens, 1837 (Limnephilinae—Drusini)	GCA_022651775.1 (Heckenhauer et al. 2022)	0.7	1/n.a.	2401	S: 95.71% D: 0.27% F: 1.92% M: 2.1%
***Philocasca rivularis*** Wiggins, 1968 (Philocascinae)	GCA_054127805.1 (this study)	**1.2**	**35.2/n.a.**	**931/n.a.**	**S: 96.75%** **D: 0.99%** **F: 0.93%** **M: 1.33%**
*Hesperophylax magnus* Banks, 1918 (Limnephilinae—Hesperophylacini)	GCA_026573805.1 (Hotaling et al. 2023)	1.2	11.2/n.a.	980/n.a.	S: 96.08% D: 1.7% F: 0.83% M: 1.39%
***Enoicyla pusilla*** Burmeister, 1839 (Limnephilinae—Limnephilini)	GCA_054127735.1 (this study)	**1**	**35.6/n.a.**	**127/n.a.**	**S: 96.62%** **D: 0.99%** **F: 0.91%** **M: 1.49%**
*Glyphotaelius pellucidus* Retzius, 1783 (Limnephilinae—Limnephilini)	GCA_936435175.1^a^ (McSwan et al. 2023)	1	8.2/36.8	284/56	S: 96.99% D: 0.51% F: 0.99% M: 1.52%
*Halesus radiatus* Curtis, 1834 (Limnephilinae—Limnephilini)	GCA_022606495.2 (Heckenhauer et al. 2022)	0.97	0.12/0.12	12,627/12,485	S: 84.82% D: 1.07% F: 6.42% M: 7.67%
*Limnephilus auricula* Curtis, 1834 (Limnephilinae—Limnephilini)	GCA_951813805.1^a^ (McCulloch et al. 2023)	0.97	8.1/34.8	229/44	S: 97.15% D: 0.43% F: 0.96% M: 1.47%
*Limnephilus lunatus* Curtis, 1834 (Limnephilinae—Limnephilini)	GCA_917563855.2^a^ (Austin et al. 2023)	1.3	19/95.4	138/38	S: 96.64% D: 0.64% F: 0.91% M: 1.52%
*Limnephilus marmoratus* Curtis, 1834 (Limnephilinae—Limnephilini)	GCA_917880885.1^a^ (Clifford et al. 2023)	1.6	8/56.2	394/67	S: 96.72% D: 0.53% F: 0.91% M: 1.84%
*Limnephilus rhombicus* Linnaeus, 1758 (Limnephilinae—Limnephilini)	GCA_929108145.2^a^ (Broad et al. 2023)	1.6	10.8/54.2	271/61	S: 96.56% D: 0.69% F: 1.09% M: 1.65%

*Note:* *Eubasilissa regina* was used as an outgroup in this study; in bold: this study.

^a^ Produced by The Darwin Tree of Life Project (The Darwin Tree of Life Project Consortium 2022).

### 2.5 Structural and Functional Genome Annotation

We annotated the genomes structurally using the web server interface https://www.plabipd.de/helixer_main.html of Helixer version 0.3.4 (Stiehler et al. 2020; Holst et al. 2023) with the lineage‐specific mode set to “invertebrate.” We extracted peptides from the resulting gff files using gffread 0.11.4 (Pertea and Pertea 2020) using option *‐y* to write a protein fasta file with the translation of coding DNA sequence for each record. Completeness of the annotation was assessed with compleasm *protein* using the Endopterygota lineage dataset. For functional annotation, predicted proteins were searched against the ncbi‐blast protein database using ncbi‐blast 2.10.0 *blastp* (Altschul et al. 1990) with an e‐value cutoff of 10–4 and –max_target_seqs set to 10. To make the Blast search more efficient, before running *blastp*, we splitted the amino acid fasta with the predicted proteins from helixer into multiple files with 50 sequences each using awk and created a *blastp* job array. The resulting xml files were concatenated into one single file using *cat*. We used the command line version of Blast2GO 1.4.4 (Conesa and Götz 2008) to assign functional annotation and GO terms to the predicted proteins.

### 2.6 Analyses of Transposable Elements

We analyzed the transposable elements (TE) in comparison with all available Limnephilidae genome assemblies (Table 1). Transposable elements were identified, curated, and annotated using the fully‐automated pipeline Earl Grey 4.2.4 (Baril et al. 2024) with optional parameters ‐*r == RepeatMasker search term: arthropoda* and *‐d == Create soft‐masked genome at the end: yes*. We compared the resulting TE classifications and repeat proportions across Limnehilidae (see Appendix S1). In addition, we compared repeat landscape plots. These summarize relative TE activity by illustrating relative abundance of repeat classes in the genome against the Kimura 2‐Parameter divergence from the consensus and are used to examine if the observed abundance patterns of specific TEs are driven by shared ancient proliferation events (moderate to high divergence from consensus) or recent/ongoing TE activity (low divergence from consensus).

### 2.7 Species Tree Reconstruction

We used the predicted single copy orthologs of Limnephilidae species (Table 1) obtained by compleasm (explained above) to infer phylogenetic trees using two different approaches. In the first approach, we concatenated single copy ortholog alignments and subsequently estimated a species tree using maximum likelihood. In the second approach, we first inferred maximum likelihood trees for each locus (single copy ortholog) and then used a multispecies coalescent approach to estimate a species tree. For both approaches, we aligned amino acid sequences of each single‐copy ortholog from each species with MAFFT 7.520 with the L‐INS‐i algorithm (Rozewicki et al. 2019). Alignments were cleaned using Aliscore 02.2 and Alicut 2.31 (Misof and Misof 2009). For the concatenation approach, we first concatenated the cleaned alignments into a supermatrix using FASconCAT 1.11 (Kück and Meusemann 2010). We then inferred a phylogenetic tree with maximum likelihood using IQ‐TREE 2.1.3 (Minh et al. 2020) using the option ‐m TESTMERGEONLY to select the best fitting partition scheme and using ModelFinder (Kalyaanamoorthy et al. 2017) to choose the optimal protein models for each metapartition (m MFP). We performed 1000 bootstrap replicates and optimized ultrafast bootstrap trees by applying nearest neighbor interchange based on bootstrap alignments (bnni option).

For the coalescent approach, we generated an individual gene tree (maximum likelihood) for each locus with IQ‐TREE using option ‐m MFP to determine the best‐fit models for each gene tree and using 1000 bootstrap replicates to assess branch support. We then generated the species tree from the estimated gene trees using ASTRAL‐III v.5.7.1 (Zhang et al. 2018) with default settings. *Eubasilissa regina* McLachlan, 1871 (family Phryganeidae; Phryganeinae) was used as an outgroup. We visualized the trees using FigTree v.1.4.4 (http://tree.bio.ed.ac.uk/software/figtree/, last accessed 2025‐20‐22).

### 2.8 Identification and Annotation of Heavy‐Chain Fibroin

We identified *h‐fibroin* genes in the newly produced primary and alternate assemblies by using tBLASTn to search the assemblies with the conserved n‐ and c‐termini of *Hesperophylax occidentalis* Banks, 1908 (Ashton et al. 2013; Heckenhauer et al. 2023; Hotaling et al. 2023) in Geneious Prime 2022.1.1 (https://www.geneious.com) with default settings. We extracted the region of the blast hits plus 10,000 bp of flanking regions from the assembly using the sequence view “extract” in Geneious and annotated this region using Augustus 3.3.3 (Hoff and Stanke 2019). We translated protein‐coding nucleotide sequences of the *h‐fibroin* with the Geneious Tool “Translate” using the standard genetic code and predicted signal peptides with the SignalP 6.0 server (Teufel et al. 2022) using the following settings: organism = Eukarya, model mode = slow. To examine the amino acid composition of each h‐fibroin protein sequence, we used ExPASy ProtParam (https://web.expasy.org/protparam/, last accessed 2025‐10‐23; Gasteiger et al. 2005). H‐Fibroin sequences were compared to previously published h‐fibroins of aquatic Trichoptera (Heckenhauer et al. 2023), as well as aquatic and terrestrial Lepidoptera (Heckenhauer et al. 2024), see Appendix S2.

### 2.9 Scanning Electron Microscopy (SEM) Imaging and Energy Dispersive X‐Ray Spectroscopy (EDS)

To determine whether the silk of *P. rivularis* was phosphorylated (as has been previously determined for aquatic caddisfly silk), we performed elemental analysis of the *P. rivularis* silk using an Apreo C SEM (ThermoScientific, Waltham, Massachusetts, USA) equipped with an Energy Dispersive X‐ray detector (EDAX Silicon‐drift XEDS, AMETEK, Berwyn, Pennsylvania, USA) at the Brigham Young University Electron Microscopy Facility. The larvae were removed from the cases and placed with glass beads as substrate for case construction. Cases were collected in May and July of 2023 and stored in ultra‐pure water or dry at 4°C. Case fragments were placed on carbon mounted on metal stubs. Topographic SEM imaging of the case fragments were performed without a metal coating using low vacuum and angle‐selective backscattered detectors under low pressure conditions. Non‐quantitive elemental analysis was conducted on the same samples to examine the presence of Phosphorous. Point and 2D maps captured the silk fibers and glass beads for elemental background contributions.

## 3 Results

### 3.1 Description of the Genomes

#### 3.1.1 Sequencing Coverage and Quality of Genome Assembly and Annotation

**FIGURE 1:**
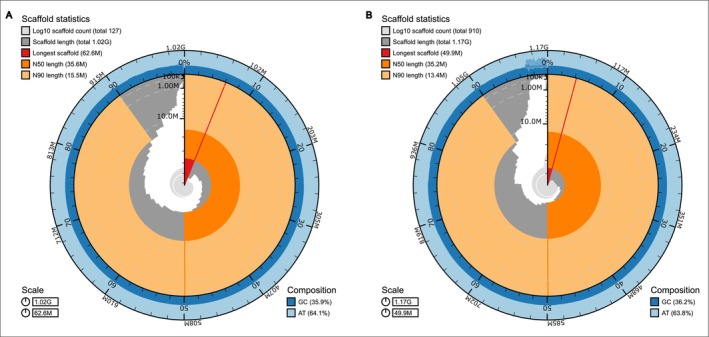
Snail plots representing genome statistics for *Enoicyla pusilla* (A) and *Philocasca rivularis* (B) generated using BlobToolKit (Challis et al. 2020). The red segment represents the longest scaffold. The other scaffolds (dark gray) are arranged in size‐order moving clockwise around the plot starting from the outside of the central plot; dark orange arcs: N50 values, light orange arc: N90 values; light gray spiral: cumulative scaffold count with a white line at each order of magnitude; dark versus light blue area: mean GC versus AT content at 0.1% intervals.

In this study, we generated whole genome assemblies for two Trichoptera species that are found in terrestrial environments, *E. pusilla* and *P. rivularis* , using PacBio HiFi sequencing. The genome assemblies generated here represent the first genomic resources for the genera *Enoicyla* and *Philocasca* and in general for terrestrial caddisflies. For *E. pusilla*, sequencing resulted in 4,160,201 HiFi reads (total 40.97 Gbp, ~ 47× sequencing coverage) with a read N50 of 12,510 and an average read length of 9848 bp. Genomescope2 predicted a genome length of 872.89 Mbp with 63.9% unique sequence (Figure S1). FCS detected one contamination with *Hydra vulgaris* (Cnidaria: Hydrozoa) and 50 contaminations with *Rickettsia* (microbial endosymbiont). These were removed from the genome assembly. For *P. rivularis* , 6,244,708 reads (86.6 Gbp, ~72× sequencing coverage) were obtained. The read N50 was 14,068 and the average read length was 13,874 bp. Genomescope2 estimated a genome size of 910.11 Mbp with 66.6% unique sequence (Figure S2). FCS detected adaptors in two contigs and contaminations in 3594 contigs. These were mostly prokaryotes (3580) such as CFB group bacteria and proteobacteria, but also included viruses (3), plant (1 moss), algae (2), and alveolates (8). The two contigs were trimmed for adaptor sequences and contaminated contigs were removed from the genome assembly. Various statistics representing contiguity and completeness were measured to evaluate the quality of the genome assemblies. The contamination‐free assemblies were of high quality with respect to contiguity and ortholog completeness. The assembly length of *E. pusilla* is 1,016,837,082 bp and consists of 127 contigs with a contig N50 of 35,602,009. Compleasm analysis recovered 97.61% complete orthologs of which 96.62% are single‐copy (Figure 1A, Table 1). The assembly length of *P. rivularis* is 1,169,981,465 bp and consists of 910 contigs with a contig N50 of 35,208,318. Compleasm analysis recovered 97.74% complete orthologs of which 96.75% are single‐copy (Figure 1B, Table 1).

#### 3.1.2 Structural and Functional Genome Annotation

Structural annotations resulted in the prediction of 18,686 and 18,961 proteins in *E. pusilla* and *P. rivularis* , respectively. Compleasm analysis revealed ortholog completeness of 83.99% and 86.86% for the *E. pusilla* and *P. rivularis* annotations, respectively. Of the annotated proteins in *E. pusilla*, 90% had BLAST hits, 73% were assigned GO terms, and 40.1% were functionally annotated with Blast2GO. Of the annotated proteins in *P. rivularis* , 88.26% returned Blast hits, 72.62% were assigned GO terms, and 41.28% were functionally annotated with Blast2GO. The major biological processes found in the two genomes are cellular and metabolic processes. Binding and catalytic activity are the largest subcategories in molecular function. Regarding the cellular component category, most proteins were assigned to cellular anatomical entities.

#### 3.1.3 Repetitive Element Dynamics

**FIGURE 2:**
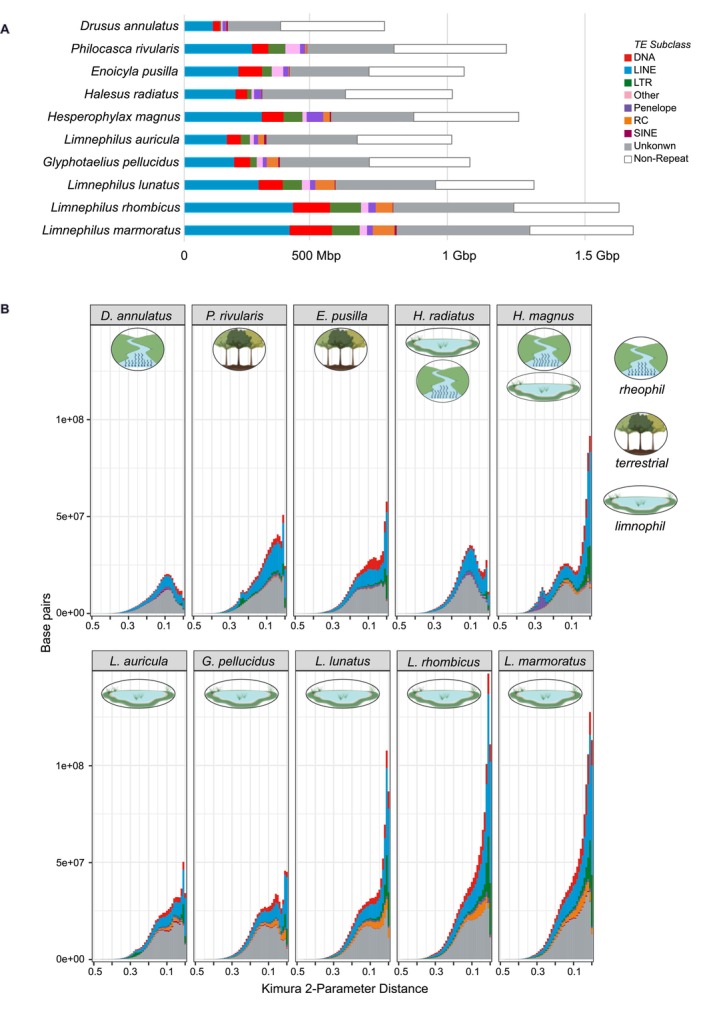
Repeat dynamics in Limnephilidae. (A) Assembly length and repetitive DNA content in Limnephilidae species. The total length of bars indicates assembly size and colored segments within bars indicate the fraction of the assembly belonging to major TE subclasses identified with Earl Grey 4.2.4. Other: Simple Repeat, Microsatellite, RNA. (B) Repeat landscape plots, where the *x* axis indicates divergence from consensus in Kimura distance, and the *y* axis indicates the percentage of the genome annotated as TE for each level of divergence. Ancient activity (greater divergence to consensus) appears on the left‐hand side, while more recent activity is shown towards the right (greater similarity to consensus). Taxa are arranged according to phylogeny (Figure 3) and focal habitat: terrestrial, lotic (i.e., preferentially in running waters), lentic (i.e., preferentially in standing waters) and generalist (i.e., no clear current preference). (B) Repeat abundance and classification in 10 Limnephilidae genomes. The number of bp for each repeat type is given for each caddisfly genome. Habitat icons were created in BioRender. Heckenhauer, J. (2026): https://BioRender.com/6a2o0kx.

Previous studies found expanded genomes with high amounts of transposable elements (TEs) in caddisfly clades with higher ecological diversity. We found that the amount of repetitive DNA across Limnephilidae is high and comprises 787 Mbp on average, or a mean genome proportion of 66.22% (for details see Appendix S1). However, we detected differences across Limnephilidae species with different habitat preferences. The lowest percentage of repeats was detected in *Drusus annulatus* Stephens, 1837 (48.09%, Figure 2A) which has a lotic habitat focus, it occurs in running waters. Highest percentage of repeat content (71.89%–76.98%) was observed in three *Limnephilus* species ( *Limnephilus lunatus* Curtis, 1834, *Limnephilus marmoratus* Curtis, 1834, *Limnephilus rhombicus* Linnaeus, 1758, Figure 2A). Interestingly, repeat content was lower in *Limnephilus auricula* Curtis, 1834 and *Glyphotaelius pellucidus* Retzius, 1783 (both > 65%, Figure 2A). The focal habitat of these five species is lentic, they preferentially occurr in standing waters. The two species associated with terrestrial habitats studied here have a repeat content of 65.14% ( *P. rivularis* ) and 66.01% (*E.pusilla*). While a majority of the repeats are TEs, only a small portion was classified as simple repeat, microsatellite or RNA (range 0.77%–4.57%, Figure 2B: pink). A large fraction of repeats remained unclassified (range: 26.02%–33.79%, Figure 2B: gray). Unclassified repeats likely present repetitive elements specific for Trichoptera that are not represented in repeat databases preventing efficient annotation, a problem that has been reported for most non‐model insect groups (Sproul et al. 2023). Of the classified repeats, long interspersed nuclear elements (LINEs) are the most abundant and comprise 20.2% on average (range: 14.58%–25.15% Figure 2B: blue), followed by DNA transposons (3.46%–9.41%, Figure 2B: red). Interestingly, the proportion of the genome that consists of DNA transposons in *E. pusilla* is high (8.46%, mean 6.36%). However, we observed similar differences in other repeat classes between Limnephilidae species indicating a dynamic evolution of repeats across this family. As previously reported, major expansions of TEs contribute to larger genomes in Trichoptera (Heckenhauer et al. 2022). For example, *D. annulatus* exhibits a genome size of 728 Mbp of which only less than half (350 Mbp) consists of repeats (Figure 2), while genome size of *L. marmoratus* is 1.63 Gbp, of which more than three‐quarters (1.25 Gbp) consist of repeats.

In all species, the majority of the classified repeats were LINEs and DNA transposons. Our TE age distribution analysis indicates that the high abundance of LINEs and DNA transposons was caused by ongoing/recent activity occurring independently across Limnephilidae rather than by shared, ancient bursts of TEs. The pattern that TEs of most Limnephilidae species studied (especially *L. lunatus* , *L. rhombicus* , *L. marmorata* , *H. magnus* , *G. pellucidus* , and *E. pusilla* ) exhibit low Kimura 2‐Parameter divergences, particularly LINEs, DNA transposons, and long terminal repeats (LTRs) (Figure 2B: peaks towards the right). This is in line with the general results of Heckenhauer et al. (2022). Although short interspersed nuclear elements (SINEs) are not abundant across Limnephilidae, we find evidence of shared ancient bursts of SINE activity in most Limnephilidae species (especially in *D. annulatus*, Figure 2B: peaks towards the left). However, we find variations from these general patterns in single Limnephilidae species. For example, we find signals of ancient proliferation events of LTR activity in *P. rivularis* and *auricula*, of penelope (PLE) activity in *H. magnus* and of rolling circle (RC) activity in *L. auricula* and *H. magnus* . In *G. pellucidus* and *L. lunatus* , *L. rhombicus* , *L. marmorata* activity of RCs is ongoing/recent. For repeat landscape plots separated by TE subclasses, see Figure S3.

#### 3.1.4 Species Tree

**FIGURE 3:**
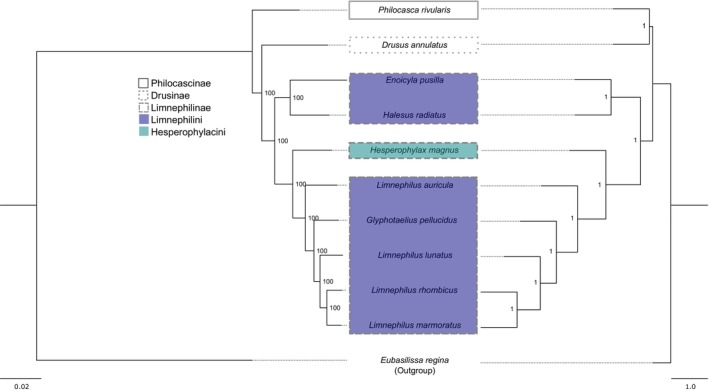
Phylogenetic trees of Trichoptera based on single copy orthologs based on concatenation (left) and multi‐coalescent (right) approach. Contour patterns of boxes indicate subfamilies Philocascinae (dashed), Drusinae (pointed), and Limnephilinae (continuous). Tribal classifications within Limnephilinae are color coded: Lilac: Limnephilini, mint: Hesperophylacini. Node labels represent bootstrap values (left) and local posterior probabilities (right). *Eubasilissa regina* (family Phryganeidae) was used as an outgroup.

To explore the phylogenetic relatedness of the two Limnephilidae species with terrestrial habitats within Limnephilidae, we estimated phylogenetic trees using single copy orthologs of all Limnephilidae species with available genomes using two different methods (concatenation/supermatrix and summary/coalescent approach). Both methods resulted in highly supported phylogenetic trees (all nodes support values of 1 or 100). The trees were largely congruent, except for the position of *P. rivularis* (subfamily Philocascinae). In the tree obtained by the supermatrix approach, *P. rivularis* sits on a single branch sister to the rest of the species sampled (Figure 3: left). In the tree resulting from the multispecies coalescent approach, *P. rivularis* forms a clade with *D. annulatus* (subfamily Drusinae). This clade is sister to all other species included in this study (Figure 3: right). Moreover, our analyses showed that *E. pusilla* forms a clade with *H. radiatus* (both subfamily Limnephilinae, tribe Limnephilini). This clade is sister to the remaining taxa consisting of the Limnephinae tribes Hesperophylacini and Limnephilini. The trees obtained in this study are also congruent with previously published phylogenetic hypotheses, in the sense that Drusinae is sister to Limnephilinae and Hesperophylacini is nested within Limnephilini (e.g., Frandsen et al. 2024), rendering this tribe paraphyletic. Interestingly, *L. auricula* , which has not been included in previous phylogenetic studies, appears separated from the other *Limnephilus* species, as *G. pellucidus* (also belonging to subfamily Limnephilinae, tribe Limnephilini) is nested between the clade containing the *Limnephilus* species.

#### 3.1.5 Amino Acid Composition of the Major Silk Gene H‐Fibroin and Elemental Analysis of the Silk

**FIGURE 4:**
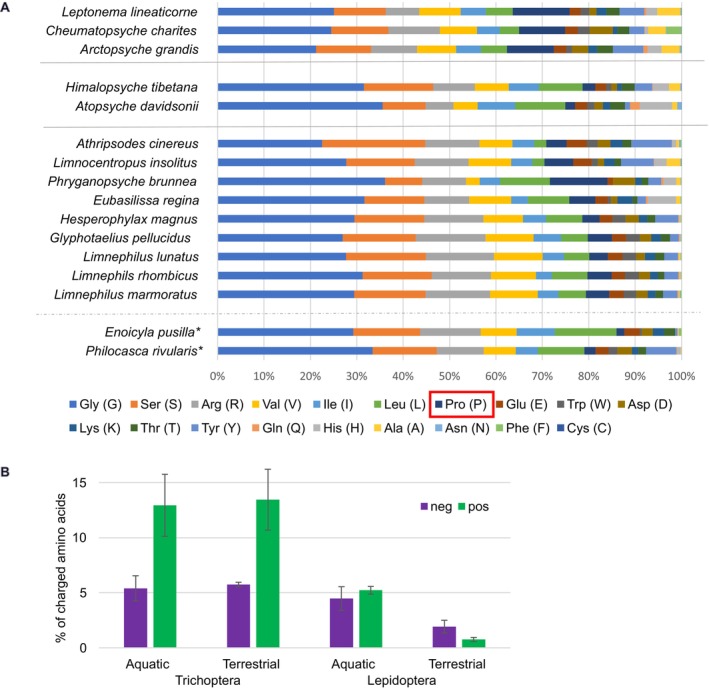
(A) Amino acid composition of h‐fibroins of Trichoptera species with different silk usage (separated by gray lines). From top to bottom: Retreat/capture net makers, cocoon‐makers, case makers. *Terrestrial case‐maker species examined in this study; (B) Mean amino acid composition (%) of negatively (purple) and positively charged (green) amino acids of full‐length h‐fibroins of aquatic (14 species) and (semi‐)terrestrial (2 species) Trichoptera, as well as aquatic (4 species) and terrestrial (5 species) Lepidoptera.

**FIGURE 5:**
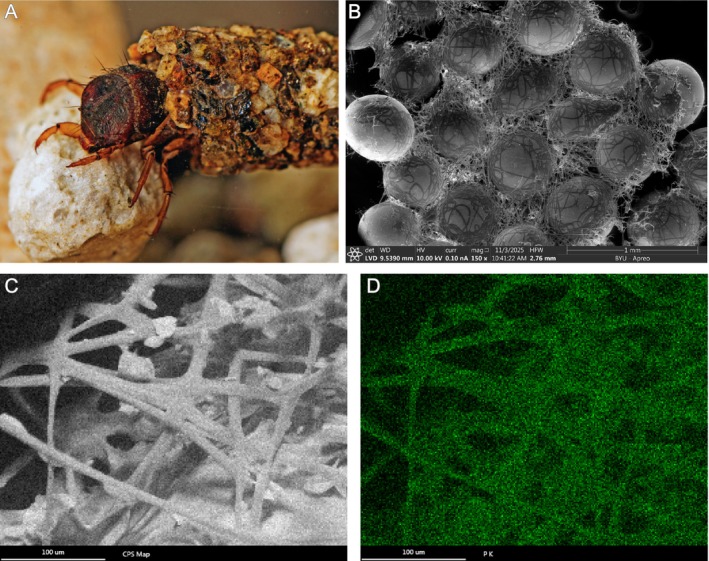
*Philocasca rivularis* silk. (A) *Philocasca rivularis* fifth instar larva with sand case. Photo taken by Greg Courtney (Iowa State University). (B) Topographical micrographs of *P. rivularis* silk case fragments constructed with glass beads. The case sample was stored dry. Images acquired under low pressure conditions using the LVD detectors at 10 kV (all), 0.10 nA. Scale bars indicate width. (C, D) SEM‐EDS analysis of *Philocasca rivularis* silk case fragments constructed with glass beads. SEM of field of view, counts per second Map (C); Phosphoros 2D map (D).

*E. pusilla* uses silk and sand grains and/or litter fragments encountered in their habitats to construct portable tube cases (Rathjen 1939; Harding 1998). The cases of *P. rivularis* are constructed of sand grains bound with silk (Figure 5A,B). Trichoptera diverged from an ancestor shared with primarily terrestrial sister order Lepidoptera (moths and butterflies) within superorder Amphimenoptera around 290 million years ago (Misof et al. 2014). In Amphismenoptera, the major component of silk proteins is encoded by the *h‐fibroin* gene. To find evidence for adaptations of silk to terrestrial versus aquatic environments, we identified the *h‐fibroin* gene and h‐fibroin protein sequences of the two caddisflies species associated with terrestrial habitats and compared these to the h‐fibroin sequences of previously published Amphismenoptera species. The full‐length *h‐fibroin* genes of the two species occurring in terrestrial habitats exhibit the typical overall structure. First, they show a similar organization of introns and exons: a short and a long exon are separated by a single intron, leading to a total length of 25,611 and 27,220 bp in *E. pusilla* and *P. rivularis* , respectively (Table 2). Second, the *h‐fibroin* exhibits a central repetitive region consisting of repeat modules. This region is embedded between conserved termini and transition regions. The transition regions show similarities to the repeat modules but their sequence is unique, for example, they occur only one time in the *h‐fibroin* (Notes S1 and S2). Third, heterozygosity of the *h‐fiborin* alleles within individuals, which was first reported by Frandsen et al. (2023), was detected in both species (Table 2) with the alternate alleles being < 5% shorter (24,747 and 27,394 bp in *E. pusilla* and *P. rivularis* , respectively) than then primary alleles. In contrast to the conservation of overall structure of the *h‐fiborin*, sequence content of the central repetitive region varies widely within Trichoptera (Standring et al. 2025). In general, each repeating structural module of the h‐fibroin protein contains a region of (SX)nE (S: serine, X: aliphatic acid or arginine, n: number of SX motif, usually 2–6 times, E: glutamic acid, Heckenhauer et al. 2023). While these regions are interspersed with glycine‐rich regions in most cocoon and case makers, retreat makers exhibit glycine–proline‐rich regions. The glycine‐(proline) rich regions are usually variable in length (Heckenhauer et al. 2023). We observed a typical tube‐case maker pattern of repeat modules in the two study species. In *E. pusilla* , the central region of the h‐fibroin consists of three repeat modules (RM, for examples see Table 3 and Note S1). RM1 consists of a (SX)4E[11](SX)4E motif and glycine‐rich region (26–75 residues), RM2 and RM3 consist of a single (SX)4E motif and glycine‐rich region (14 residues). RM1 is repeated 90 times, while RM2 and RM3 are repeated 18 and 23 times, respectively. In *P. rivularis* , the central region of the h‐fibroin consists of five repeat modules (for examples see Table 3 and Note S2). Each repeat module consists of a (SX)_4_E[14](SX)_4_E[11](SX)_4_ motif and a glycine‐rich region (21–112 residues). Regarding the amino acid compositions of the h‐fibroins, as expected, the percentage of proline is lower (1.7% and 2.4% for *E. pusilla* and *P. rivularis* respectively, Figure 4A) than in retreat and capture net making species (Annulipalpia) and in *Phryganopsyche brunnea* Wiggins, 1969 whose case resembles that of a retreat‐makers in the sense that it bends under its own weight when removed from the water (mean: 11.18%, Heckenhauer et al. 2023; Deng et al. 2025; Appendix S1). Interestingly, it is even lower than in aquatic species with rigid tube cases (mean: 4.91%, Appendix S1), but similarly low as in cocoon‐makers (mean: 2.4%, Appendix S1). In addition, the amount of leucine is high in both terrestrial species (Figure 4A, 13.3% in *E. pusilla* , 10% in *P. rivularis*), a pattern observed in cocoon‐making species (mean: 10.1%), as well as in *P. brunnea* (10.7%). Higher amounts of charged amino acids have been detected in the h‐fibroin of aquatic Lepioptera compared to terrestrial species (Heckenhauer et al. 2024). However, the percentages of charged amino acids of the h‐fibroin of the two species studied here are similarly high as those of aquatic species (negatively charged 5.9% and 5.6%, positively charged: 15.5% and 11.5%, for *E. pusilla* and *P. rivularis* respectively, Figure 4B).

**TABLE 2:** Full‐length *h‐fibroins* of the two terrestrial caddisfly species.

Species	Alelle	Gene length	CDS	Exon 1	Intron 1	Exon 2	Protein
*E. pusilla*	prim.	25,611	24,771	42	840	24,729	8257
	alt.	24,747	23,907	42	840	23,865	7969
*P. rivularis*	prim.	27,220	26,352	42	868	26,310	8784
	alt.	27,394	26,064	42	1330	26,022	8688

*Note:* Gene length including exons and introns, length of coding DNA sequence (CDS), size of exons, and introns are given in bp. Protein sizes are given in amino acids.

Abbreviations: alt., alternate assembly; pri., primary assembly.

**TABLE 3:** Repeat motifs (RM) occurring in the h‐fibroin protein sequence of the two terrestrial caddisfly species.

RM	Pattern	Example	No.
***E. pusilla***			
RM1	(SX)_4_E[11](SX)_4_ GP‐rich[26–75]	SISRSVSIERIVTPGSITKISRSSSVSIE VGRRGGLGGLGGLQGIGGLRGLGGRRGKV	90
RM2	(SX)_4_E GP‐rich[14]	SESFSVSIE RGIRRGPWGRRGKV	18
RM3	(SX)_4_E GP‐rich[14]	SGSFSVSIE RGIRRGPWGRRGKV	23
***P. rivularis***			
RM1	(SX)_4_E[14](SX)_4_E[11](SX)_4_E GP‐rich[21]	SGSVSVSIERGIRRGPWGRRGKVSISRSVSIERIVTPGVYTHISRSSSVSVE GGRRRGPWGYGRGLGGYGVGI	1
RM2	(SX)_4_E[14](SX)_4_E[11](SX)_4_E GP‐rich[47–92]	SGSLSVSVERSYRRGPWGRRGKVSISRSVSVERIVTPGVYTHISRSSSVSVE GGRRGGLWGYGRGLGGLSGSGDLDGLGGYGGLGGLGGYGGLGGYGGI	2
RM3	(SX)_4_E[14](SX)_4_E[11](SX)_4_E GP‐rich[21–53]	SGSLSVSVERSYGRGPWGRRGKVSISRSLSIERIVSPGVYTHISRSSSVSVE GGRLGGPWGYGRGLGGYGVGI	6
RM4	(SX)_4_E[14](SX)_4_E[11](SX)_4_E GP‐rich[62–100]	SGSLSVSVERGYRRGPWGRRGKVSISRSVSIERIVTPGVYTKISRSSSVSIE GGRRGGLWGYGRGLGGLSGSGDLDGLGGYGGLDGYGGLGGLGGYGGL GGLGGYGGPGGYGGI	16
RM5	(SX)_4_E[14](SX)_4_E[11](SX)_4_E GP‐rich[21–112]	SGSLSVSVERSYRRGPWGRRGKVSISRSVSIERIVTPGVYTKISRSSSVSVE GGLRRGPWGYGRELGGYGVGI	47

Moreover, phosphorylation of serines and its complexation with metal ions acquired from the aquatic environment has been detected in analyses of silk of aquatic caddisfly species, leading us to question whether (semi‐)terrestrial silk would maintain this adaptation thought to be central to aquatic silks. Using EDS, we generated two‐dimensional element maps of naturally spun silk of *P. rivularis* . We found evidence for the presence of Phosphorus co‐localized with the silk fibers at concentrations above background level (Figure 5C,D). This indicates that phosphorylation plays an important role in species associated with terrestriality as well.

## 4 Discussion

This study provides the first genome assemblies and annotations for two caddisfly species associated with terrestrial habitats. The two assemblies presented in this study are characterized by high contiguity (contig N50 > 35, Figure 1) and completeness (> 96% single BUSCOs) and are thus of higher or comparable high quality as previously published caddisfly genomes (Table 1). Genome size predicted by Genomescope2 is slightly lower than the actual assembly length for both species. In highly repetitive genomes, this has been observed before (Austin et al. 2017; Sánchez‐Herrero et al. 2019; Heckenhauer et al. 2019; Deng et al. 2025) since *k‐mer* estimates used by Genomescope2 are prone to underestimate repetitive element content, resulting in smaller genome size estimates.

Generally, TE insertions are considered to have deleterious effects on their host's fitness activity (e.g., Mackay 1989) and are thus kept at low frequencies by purifying selection (Charlesworth and Langley 1989). On the other hand, TE activity can also be a critical source of new genetic variation potentially driving diversification via chromosomal rearrangements and transposition events (reviewed by Tossolini et al. 2025).

Previous broad scale studies detected large genome size differences within different clades of Trichoptera (Olsen et al. 2021) and found that TE expansions are important drivers of large caddisfly genome sizes (Heckenhauer et al. 2022). Large genome sizes and TE expansions preferentially evolved in tube case making caddisfly clades that exhibit higher ecological diversity, such as Limnephilidae. Specifically, in a study using quantified TE presence in BUSCO genes as a surrogate measure for the evolution of TEs within and around protein‐coding genes (Heckenhauer et al. 2022) showed a linear relationship of TE‐BUSCO gene associations and increasing genome size and in tube case makers including Limnephilidae (Heckenhauer et al. 2022).

When comparing the proportion of the genome that consists of repeats across Limnephildae, we found that it ranges from 48.09% to 76.98%. The genome with the lowest proportion of repeats was that of *D. annulatus* (< 48%, Figure 2A). This species has a lotic habitat focus. Proportion of repeat content in species with a lentic habitat focus, ranged from > 65% in *L. auricula* and *G. pellucidus* to 72%–75% in the remaining *Limnephilus* species (Figure 2A). Lentic environments (e.g., ponds, lakes, marshes) are more variable in temperature and oxygen availability than small to mid‐sized rivers. Thus, lentic species might be exposed to greater environmental variation as a potential source of stress which might lead to more frequent TE bursts and reduced efficacy of natural selection in purging deleterious effects of TE expansions (Heckenhauer et al. 2023). Further, TEs have been shown to interact with protein coding genes and can affect gene expression, rates of evolution, and chromosome structure. It has been proposed that TEs have been important for the evolution of gene regulation as they can act as genome‐wide sources of regulatory elements and activities (as reviewed in Feschotte 2008; Chuong et al. 2017). Epigenetic silencing (e.g., heterochromatin formation) mechanisms suppress the activity of TEs (Slotkin and Martienssen 2007). Repressive marks can occur near regulatory gene regions and spread to adjacent sequences (Lee and Karpen, 2017; Wei et al. 2022). TE sequences can be co‐opted to form gene regulatory networks with genome‐wide effects on gene expression (Chuong et al. 2017). Also, changes in chromatin accessibility can lead to cooption of transposable elements into the regulatory landscape (Vrljicak et al. 2023). To sum up, by impacting transcriptional and epigenetic modifications and affecting chromatin organization, TEs can play a role in phenotype evolution and adaptation (see review Tossolini et al. 2025). For example, cichlid fish species with color markings on their fins feature a TE in the cis‐regulatory region of pigmentation genes (Santos et al. 2014); the mutation responsible for the black carbonaria morph of the peppered moth has been identified as a TE within the cortex gene (Hof et al. 2016). Our study shows that Limnephilidae are a suitable family to further investigate TEs and their role for genomic adaptations to different habitats, for example, through the modification of chromatin folding and via transcriptional activation, processes that influence the expression of neighboring genes. It is possible that elevated TE activity in the ecologically diverse Trichoptera species could facilitate more rapid evolution in genes related to habitat preferences (e.g., desiccation/thermal tolerance, respiration). More research is necessary to identify direct conclusions regarding TEs and adaptation. A next step would be to investigate the level of conservation of specific TE‐gene associations across Limnephilidae and test if certain gene family expansions/contractions, especially of genes involved in habitat shifts, are associated with TE abundances.

However, it is also possible that the TE expansions detected in this study have more to do with phylogenetic history and ongoing population dynamics. Further research is essential to examine these relationships.

Phylogenetic reconstructions using BUSCO genes from all available Limnephilidae genomes are generally in line with comprehensive phylogenomic studies of caddisflies (e.g., Frandsen et al. 2024) which also revealed that (1) subfamily Drusinae is sister to subfamily Limnephilinae and (2) that tribe Hesperophylacini is nested within tribe Limnephilini. Interestingly, *L. auricula*, which was not included in previous phylogenetic estimates, is separated from the other *Limnephilus* species. Moreover, the positions of the two species occurring in terrestrial environments were examined. Both, the concatenation and the coalescent approach, result in phylogenetic trees with high node support. However, the position of *P. rivularis* (subfamily Philocascinae) is incongruent. While it forms a clade with *D. annulatus* in the tree resulting from the multi‐species coalescent approach, it is located on a single branch, sister to the remaining taxa of this study (all subfamily Limnephilinae) in the concatenated tree.

Biological processes, as well as methodological artifacts might cause this incongruency. Processes such as hybridization (reticulate evolution) and incomplete lineage sorting can lead to gene tree discordance. The two phylogenetic approaches used in this study handle gene tree heterogeneity differently. The summary coalescent approach focuses on congruence among the individual trees and thus accounts for incomplete lineage sorting. The concatenation approach often reflects overall sequence similarity and assumes that all genes share a single evolutionary history. Thus, it might give misleading support if incomplete lineage sorting is high (Degnan and Rosenberg 2009). Both methods assume strictly bifurcating tree models which might fail to represent network‐like relationships caused by hybridization. Therefore, further studies including more taxa and additional molecular data are needed to clarify the position of *P. rivularis* . *Philocasca* was initially placed into the subfamily Limnephilinae. However, based on morphological characters Vshivkova et al. (2007) proposed a monotypical subfamily Philocascinae. Specifically, unlike in Limnephilinae, its phallocrypt sclerotized strips are located ventrolaterally (Vshivkova et al. 2007). Moreover, *Philocasca* exhibits other specific characters, not occurring in Limnephilinae as outlined in Ruiter and Mutch (2019): wings are very broad, rounded throughout, obvious color patterns are missing, mesonotal warts are absent, larvae exhibit strongly rounded anterior portion of pronotum without transverse groove, but with unique, long, flattened, scale‐like setae on head and pronotum. In both trees, *E. pusilla* and *H. radiatus* (both tribe Limnephilini), form a clade sister to the remaining taxa consisting of the Limnephinae tribes Hesperophylacini and Limnephilini. Overall, the results indicate that terrestriality in Limnephilidae evolved multiple times. Also, our results highlight the need to revise the intrafamilial relationships of Limnephilidae. Most recent studies show that current taxonomic classifications contrast with recent phylogenetic inferences (e.g., Pauls et al. 2008; Frandsen et al. 2024).

To build their cases, *E. pusilla* and *P. rivularis* use small stones and soil particles which are glued together by silk that solidifies in the air. We successfully retrieved the complete major silk protein, h‐fibroin, from the genome assemblies of the two (semi‐)terrestrial species generated in this study. When comparing the central repetitive region of the h‐fibroins responsible for the properties of the silk with those of aquatic Trichoptera, we observed similar patterns of repeat modules as well as amino acid compositions. The percentage of proline is lower than in the h‐fibroin of retreat and capture net making species. It has been shown that higher percentages of proline in silk proteins of orb web spiders can lead to enhanced extensibility of silk (Savage and Gosline 2008a, 2008b). Flexible retreats and capture nets presumably require more extensible silk, that is, to prevent breaking of strains in strong currents or when capturing small invertebrate prey. Thus, a higher proline content could be advantageous in these species and might not be necessary in species which build rigid cases, such as the two (semi‐)terrestrial species in this study. Our study supports this hypothesis on the role of proline for the silk gene phenotype. However, the genetic basis for adaptations of silk to terrestrial habitats remains unclear in this study. In previous studies, silk genes of aquatic insects have been shown to exhibit higher percentages of charged amino acids (Papanicolaou et al. 2013; Heckenhauer et al. 2024). The greater proportion of charged amino acids might lead to proteins that are less hydrophobic and therefore less prone to clumping in an aquatic environment. This might be an adaptation for aquatic silks (Papanicolaou et al. 2013). Therefore, we expected that in caddisfly species that are found in terrestrial environments, charged amino acids would not play a major role and are thus lower, for example, lower percentages of charged amino acids were observed in terrestrial vs. aquatic Lepidoptera (Heckenhauer et al. 2024). However, contrary to our expectations, we detected high amounts of charged amino acids in (semi‐)terrestrial Trichoptera. In addition, phosphorylation of the repeating (SX)*n* motif has been hypothesized as an important molecular adaptation for underwater silk spinning from the ancestral terrestrial silk (Stewart and Wang 2010; Frandsen et al. 2019). In Lepidoptera, polyalanine and/or alternating glycine–alanine motifs form β‐sheet nanodomains responsible for the semicrystalline structure of the silk (Mayen et al. 2015). In contrast, β‐sheets in caddisflies are likely formed via the bonding of dianionic phosphorylated serine residues (pSX)nE via complexation with metal cations, such as Ca^2+^ (Ashton et al. 2013). It has been hypothesized that these differences in the sequences of the repetitive region of Lepidoptera and Trichoptera h‐fibroins are linked to adaptation to water versus air (Standring et al. 2025). In this study, two‐dimensional element maps of naturally spun silk of *P. rivularis* using EDS qualitatively revealed the presence of Phosphorus. Analyses of silk of aquatic tube case making species *H. occidentalis* and net making species *Stenopsyche marmorata* Navas, 1920 and *Parapsyche elsis* Milne, 1936 (Stewart and Wang 2010; Ohkawa et al. 2013; Frandsen et al. 2019) have shown that Phosphorus occurs primarily as phosphates on serine residues. Metal ions such as Calcium and Magnesium are complexed with those phosphoserines (Stewart and Wang 2010; Ohkawa et al. 2013). Future research quantifying metal concentrations in terrestrial caddisfly silk gland lumen is important to reveal how silk folding still takes place even without the extra strength and toughness from metals usually acquired from the aquatic environment as the fibers are spun into water. In fact, metal ions that usually strengthen and toughen the silk fibers might be obtained from the terrestrial, but humid environment and damp substrates, as the fibers are spun into air. Alternatively, the terrestrial species might have the metal ions in their silk gland lumen (taken up through food) and thus do not need to uptake the ions from the environment. Both hypotheses need to be tested in a future study. Moreover, identification and comparison of accessory genes associated with silk production are needed to shed light on potential molecular adaptations of the silk to terrestrial habitats. On the other hand, our finding that terrestrial caddisfly silk retains highly charged amino acid abundance and phosphorylation might suggest that these traits are potentially phylogenetically conserved rather than adaptive. Unfortunately, there is no comparable data from other arid‐adapted insects for these traits. Thus, our interpretation remains cautious at this point.

## 5 Conclusion

Here, we present high‐quality genomes of two caddisfly species of the ecologically diverse family Limnephilidae that are associated with terrestrial habitats. The new genomes are key resources for future genomic research on Trichoptera evolution, particularly related to habitat diversification and aquatic‐terrestrial transitions, and thus have a high usage potential. For example, they can be used in comparative genomic studies, for example, to identify gene families associated with different habitats. These might span respiratory, metabolic, olfactory, vision, thermal tolerance and other genes. Genome‐wide searches for adaptive signatures (positive selection, contractions and expansion) of gene families associated with adaptation to various habitats, might shed light on the evolutionary processes behind habitat transition in Trichoptera.

## Author Contributions

**Jacqueline Heckenhauer:** conceptualization (equal), data curation (lead), formal analysis (lead), funding acquisition (equal), investigation (lead), project administration (lead), resources (equal), supervision (equal), validation (lead), visualization (lead), writing – original draft (lead), writing – review and editing (equal). **Charlotte Gerheim:** investigation (supporting), writing – review and editing (supporting). **Gabriela Jijon:** investigation (supporting), visualization (supporting), writing – review and editing (supporting). **Robert W. Wisseman:** investigation (supporting), writing – review and editing (equal). **Steffen U. Pauls:** conceptualization (equal), writing – review and editing (equal). **Paul B. Frandsen:** conceptualization (equal), formal analysis (supporting), funding acquisition (equal), investigation (supporting), project administration (supporting), resources (equal), supervision (equal), validation (supporting), writing – review and editing (equal).

## Funding

This work was supported by Alexander von Humboldt‐Stiftung, Deutsche Forschungsgemeinschaft (502865717), and United States National Science Foundation (MCB 2217155).

## Conflicts of Interest

The authors declare no conflicts of interest.

## Supporting information


**Appendix S1:** Repetitive DNA content in Limnephilidae.

**Appendix S2:** Amino acid composition of the major silk gene *h‐fibroin*.

**Table S1:** Software tools and commands used to perform analyses of this study. All files are available at National Center for Biotechnology Information (NCBI, https://www.ncbi.nlm.nih.gov/) or at https://figshare.com/s/732ad6f0f7c6a0fa0f74.
**Table S2:** Blast hit table. Primary and alternate assemblies were blasted against termini of the *h‐fibroin* of *Hesperophylax occidentalis* to identify the *h‐fibroin* in the respective assemblies.
**Figure S1:** Genomescope Profile of *kmers* derived from HiFi reads ( *Enoicyla pusilla* ).
**Figure S2:** Genomescope Profile of *kmers* derived from HiFi reads ( *Philocasca rivularis* ).
**Figure S3:** Repeat landscape plot, where the *x* axis indicates divergence from consensus in Kimura distance, and the y axis indicates the percentage of the genome annotated as TE for each level of divergence. Ancient activity (greater divergence to consensus) appears on the left‐hand side, while more recent activity is shown towards the right (greater similarity to consensus).
**Note S1:** Schematic representation of the *h‐fibroin* of *Enoicyla pusilla* .
**Note S2:** Schematic representation of the *h‐fibroin* of *Philocasca rivularis* .

## Data Availability

Sample information (*E. pusilla*: SAMN49671951; *P*. *rivularis*: SAMN49671952), raw PacBio HiFi sequencing reads (*E. pusilla*: SRR36479557; *P. rivularis* : SRR36482349), and primary assemblies (*E. pusilla*: JBPQUU000000000; *P. rivularis* : JBPQUV000000000) have been uploaded to the National Center for Biotechnology Information (NCBI, https://www.ncbi.nlm.nih.gov/). Sequences of primary and alternate *h‐fibroins* have been uploaded to GenBank (https://www.ncbi.nlm.nih.gov/genbank/) at PX673985 and PX673986 ( *E. pusilla* ) and PX673987 and PX673988 (*P. rivularis*). Alternate genome assemblies, functional and structural annotations of the genomes, mitogenomes, repeat annotations and files relevant for phylogenetic inferences are found at: https://figshare.com/s/732ad6f0f7c6a0fa0f74 (10.6084/m9.figshare.30834434), see Table S1.
