# Supplementary material for: Genomics of Caddisfly (Insecta: Trichoptera) Species Associated With Terrestrial Habitats
Source: Ecol Evol. 2026 Apr 1;16(4):e73327. doi: 10.1002/ece3.73327 (PMC13045387; doi:10.1002/ece3.73327)
Supplement: Supplementary file 3 — Table S1: Software tools and commands used to perform analyses of this study. All files are available at National Center for Biotechnology Information (NCBI, https://www.ncbi.nlm.nih.gov/) or at https://figshare.com/s/732ad6f0f7c6a0fa0f74. Table S2: Blast hit table. Primary and alternate assemblies were blasted against termini of the h‐fibroin of Hesperophylax occidentalis to identify the h‐fibroin in the respective assemblies. Figure S1: Genomescope Profile of kmers derived from HiFi reads ( Enoicyla pusilla ). Figure S2: Genomescope Profile of kmers derived from HiFi reads ( Philocasca rivularis ). Figure S3: Repeat landscape plot, where the x axis indicates divergence from consensus in Kimura distance, and the y axis indicates the percentage of the genome annotated as TE for each level of divergence. Ancient activity (greater divergence to consensus) appears on the left‐hand side, while more recent activity is shown towards the right (greater similarity to consensus). Note S1: Schematic representation of the h‐fibroin of Enoicyla pusilla . Note S2: Schematic representation of the h‐fibroin of Philocasca rivularis . [file ECE3-16-e73327-s003.pdf]

Supplementary Material

Supplementary Table S1: Software tools and commands used to perform analyses of this study. All files are available at National Center for Biotechnology Information (NCBI, https://www.ncbi.nlm.nih.gov/) or at https://figshare.com/s/732ad6f0f7c6a0fa0f74

Analysis	Software Tool	Version	module, parameters, etc.	Results
Processing of raw data				
conversion of raw reads from bam to fastq format	samtools	1.19.1	*bam2fq*<br><raw reads in bam format> \\<br>default parameters	NCBI SRA: SRR36482349 (*P. rivularis)*<br><br>NCBI SRA: SRR36479557 (*E. pusilla)*
calculation of read statistics	fast_stats.py		-i <raw_reads.fq> \\<br>-n 50 (read N of choice)	reported in manuscript main text, section results
Genome profiling				
k-mer counting	Jellyfish	2.3.0	*count*<br><raw_reads.fq> \\<br>-C (canonical representation) \\<br>-m 21 (length of mer) \\<br>-s 1000000000 (initial hash size) \\<br>-o <output file> (mer_counts.jf)	enoicyla_pusilla_reads.jf.gz<br><br>philocasca_reads.jf.gz
generating histogram of *k-mers*			*histo*<br><reads.jf> \\<br>-o <output file> \\<br>default parameters	
genome profiling (size, heterozygosity)	GenomeScope	2.0.0	*genomescope.R*<br>-i <input histogram file from jellyfish histo> \\<br>-k 21 (kmer length used to calculate kmer spectra) \\<br>-p 2 (ploidy)	Figure S1, Figure S2
Genome assembly				
haplotype-resolved *de novo* assembly using phased assembly graphs	hifiasm	0.19.5 (*P. rivularis*)<br>0.19.8 (*E. pusilla*)	<raw_reads.fq> \\<br>-o <prefix of output files> \\<br>default parameters	primary assemblies:<br><br>NCBI: JBPQUV000000000 (*P. rivularis)*<br><br>NCBI: JBPQUU000000000 (*E. pusilla)*<br><br>alternate assemblies:<br><br>enoicyla_pusilla_alternate_assembly.fa.gz<br><br>philocasca.rivularis_alternate_assembly.fasta.gz
convert assembly from hifiasm from gfa to fasta format	awk	n.a.	awk '$1 ~/S/ {print ">"$2"\n"$3}' assembly.gfa<br>> assembly.fa	
Mitochondrial genome				

mitochondrial genome assembly from PacBio high fidelity reads	MitoHiFi	3.2	-c <assembly.fasta>,<br>-f NC_026219.1.fasta<br>(close-related mitogenome in fasta format) \<br>-g NC_026219.1.gb \<br>(close-related species mitogenome in genebank format) \<br>-o 5 (organism genetic code following NCBI table: the invertebrate mitochondrial code)	enoicyla_pusilla_final_mitogenome.gb.gz<br><br>philocasca_rivularis_final_mitogenome.gb.gz
Contamination filtering				
detection of adapters	FCS	0.5.5	*run_fcsadaptor.sh*<br>--fasta-input <assembly.fasta> \<br>--output-dir <output directory> \<br>--image /fcs/fcs-0.5.0/dist/fcs-adaptor.0.5.0.sif \<br>--container-engine singularity \<br>–euk (taxonomy: eukaryotes)	reported in manuscript main text<br><br>enoicyla_pusilla_fcs_adaptor_report.txt<br><br>philocasca_rivularis_fcs_adaptor_report.txt
detection of genome contamination			*screen genome*<br>--fasta <assembly.fasta> \<br>--gx-db gxdb/all (path to gx-database) \<br>--tax-id 1271740 or 1683747 (*E. pusilla* or *P. rivularis*, NCBI taxonomy identifier) \<br>--out-dir <output_directory>	reported in manuscript main text<br><br>enoicyla_pusilla_fcs_gx_report.txt<br>enoicyla_pusilla_CONTAMINATION.fa.gz<br><br>philocasca.rivularis.fcs_gx_report.txt<br>philocasca_rivularis_CONTAMINATION.fa.gz<br>philocasca_rivularis_CONTAMINATION_review.fa.gz
filtering of genome contamination			*clean genome*<br>-i <assembly.fasta> \<br>--action-report <*.fcs_gx.report.txt> (action-report produced by *screen genome*) \<br>--contam-fasta-out <*CONTAMINATION.fasta| (fasta for EXCLUDE entries in action report) \<br>--min-seq-len 1 (minimum sequence length to keep) \<br>--output <filtered_assembly.fasta> (modified cleaned fasta)	
Quality assessment of genome assemblies				
Visualization of quality assessment of genome assemblies	BlobToolkit	5.0.2	*blobtools create*<br>--fasta <final_assembly.fasta><br><br>*blobtools add*<br>--busco <*.full_table.tsv> (table with compleasm results)<br><br>*blobtools view*<br>--view snail<br><br>*blobtools host*<br>--api-port <port_name>,<br>--hostname <host_name> ./	reported in manuscript main text, Figure 1

quality assessment of genome assemblies	QUAST	5.2.0	*quast.py* <final_assembly.fasta>, default	reported in manuscript main text, Table 1
evaluate completeness of genome assembly, inference of phylogenetic tree	compleasm	0.2.7	*compleasm.py run*<br>-l endopterygota (lineage) \<br>-a <final_assembly.fasta \<br>-o <output directory>	reported in manuscript main text, Table 1
Genome annotation				
structural genome annotation	helixer	0.3.4	web server interface<br>https://www.plabipd.de/helixer_main.html,<br>upload nucleotide sequence file (FASTA format):<br><final_assembly.fasta> \<br>select Lineage-specific mode: invertebrate \<br>enter label for GFF feature naming: <output_prefix>	enoicyla_pusilla_helixer_annotation.gff.gz<br><br>philocasca_rivularis_helixer_annotation.gff.gz
extraction of translated CDS from gff file obtained by helixer annotation	gffread	0.11.4	*gffread*<br><input_gff_from helixer> \<br>-g <final_assembly.fasta> \<br>-o <outfile_name> \<br>-y (write a protein faa file with the translation of CDS for each record)	enoicyla_pusilla_helixer_proteins.fa.gz<br><br>philocasca_rivularis_helixer_proteins.fa.gz
evaluate completeness of genome annotation	compleasm	0.2.7	*compleasm.py run*<br>-l endopterygota (lineage) \<br>-p <predicted_proteins.fasta \<br>-o <output directory>	reported in manuscript main text
split protein.faa into multiple files with 50 sequences each	awk		awk 'BEGIN {n_seq=0;} /^>/ {if(n_seq%50==0){file=sprintf("../fa/PREFIX_aa_%d.fa", n_seq);} print >> file; n_seq++; next;} { print >> file; }' < annotated_proteins.faa	enoicyla_pusilla_blastp_all.xml.gz<br><br>philocasca_rivularis_blastp_all.xml.gz
comparison of annotated proteins to protein database	ncbi-blast	2.10.0	*blastp*<br>-query <annotated_proteins.faa>, -db nr/nr_2023-12-12/nr \<br>-outfmt 5 (alignment view format: blast xml) \<br>-max_target_seqs 10 (maximum number of aligned sequences to keep) \<br>-evalue 1e-4 (expectation value threshold for saving hits) \<br>-out <*.xml> (output_filename)	
functional genome annotation	Blast2GO	1.4.4	-tempfolder <temporary_folder> \<br>-properties cli.prop \<br>-loadfasta <annotated_proteins.fasta> \<br>-loadblast <blastp_results.xml> \<br>-mapping \	enoicyla_pusilla_b2g.pdf.gz<br><br>enoicyla_pusilla_b2g.pdf.gz

			-annotation \ -savebox <*.box> (output directory) \ -savereport <*_b2g.pdf> (output *.pdf file) \ -saveannot <*.anno> (output *.anno file)	
annotation of transposable elements	Earl Grey	4.2.4	*earlGrey*<br>-g <final_assembly.fasta> \ -s <output_prefix> (species name) \ -r arthropoda (RepeatMasker search term) \ -d yes (create softmasked genome at the end) \ -o <output_directory>	reported in manuscript main text, Figure 2, Figure S3, Appendix_S1_Repetitive_DNA.xlsx, earl_grey results for each species: *_earlgrey_summaryFiles.zip:<br>**-families.fa.strained*<br>**.familyLevelCount.txt*<br>**.filteredRepeats.bed*<br>**.filteredRepeats.gff*<br>**.highLevelCount.txt*<br>**.softmasked.fasta*<br>**.summaryPie.pdf*<br>**_classification_landscape.pdf*<br>**_combined_library.fasta*<br>**_split_class_landscape.pdf*<br>**_superfamily_div_plot.pdf*
creating new unaligned FASTA files for each single copy ortholog obtained by compleasm analysis (gene_marker,fasta) & list resulting files	awk	n.a.	awk 'BEGIN{RS=">"; FS="\n"} {m=(split($1,b,"_")); fnme=b[1]".fas";\ n=split(FILENAME,a,"/"); print ">" a[2] "\n" $2 >> fnme; close(fnme);}'\ ../*fasta<br><br>sed -i -e 's/.fasta//g' *fas<br><br>ls *fas > locus_names.txt	locus_names.txt.gz<br>FcC_smatrix.fas.gz<br>FcC_info.xls.gz
alignment of the single copy orthologs	MAFFT	7.520	locus=$(head -n $SLURM_ARRAY_TASK_ID locus_names.txt | tail -n1)<br><br>*mafft* --maxiterate 1000 --localpair ${locus} > `basename ${locus} .fas`_aligned.fas	
list of aligned loci & masking of sequence alignments	Aliscore	02.2	*aligned.fas > aligned_loci.txt<br><br>locus=$(head -n $SLURM_ARRAY_TASK_ID aligned_loci.txt | tail -n1)<br><br>perl Aliscore.02.2.pl -i ${locus} -N	
cut out noisy regions of your alignments	Alicut	2.31	default	
concatenation of alignment	FASconCAT	1.11	default	
creation of partition file	extract_partition		python3 extract_partition_file_from_fcc.py FcC_info.xls	partition_def.txt.gz

<table>
<tr><td></td><td>_file_from_fcc.
py</td><td></td><td></td><td></td></tr>
<tr><td>partition scheme search</td><td>IQ-TREE</td><td>2.1.3</td><td>iqtree2
- s FcC_smatrix.fas (alignment file) \
-spp partition_def.txt \
-safe (safe likelihood kernel to avoid numerical underflow) \
--prefix trichoptera_partition (prefix for outputfiles) \
-m TESTMERGEONLY (standard model selection) \
-mset LG+G (model used)</td><td>lim_partition.best_scheme.nex.gz</td></tr>
<tr><td>tree estimation from concatenated supermatrix</td><td>IQ-TREE</td><td>2.1.3</td><td>iqtree2
-s FcC_smatrix.fas (alignment file) \
-spp lim_partition.best_scheme.nex \
--safe (safe likelihood kernel to avoid numerical underflow) \
--prefix lim_concatenated (prefix for outputfiles) \
-m MFP (extended model selection followed by tree inference) \
-bb 1000 (replicates for bootstrap + ML tree + consensus tree) \
-bnni (pptimize UFBoot trees by NNI on bootstrap alignment)</td><td>lim_concatenated.treefile</td></tr>
<tr><td>estimation of individual locus trees & concatenation of the trees</td><td>IQ-TREE</td><td>2.1.3</td><td>ls *aligned.fas > locus_alignments.txt

align=$(head -n $SLURM_ARRAY_TASK_ID locus_alignments.txt | tail -n1)

iqtree2
-s $align (alignment file) \
-m MFP (extended model selection followed by tree inference) \
-bb 1000 (replicates for bootstrap + ML tree + consensus tree) \
-pre `basename $align _aligned.fas` (prefix)

cat *treefile > all.tre</td><td>locus_alignments,txt
all.tree.gz</td></tr>
<tr><td>estimation of species tree</td><td>ASTRAL-III</td><td>5.7.1</td><td>astral.5.7.1.jar
-i all.tre (input) \
-o astral.tre (output)</td><td>astral.tree</td></tr>
<tr><td>visualization of trees</td><td>FigTree</td><td>1.4.4</td><td>n.a.</td><td>Figure 3</td></tr>
<tr><td colspan="5">h-fibroin identification and amino acid composition</td></tr>
<tr><td>search of assemblies against the</td><td>tBLASTn</td><td></td><td>default</td><td>Table S2</td></tr>
</table>

conserved n- and c-termini of *Hesperophylax occidentalis*				
annotation of region of blast hits (see table S2) and 1,000 bp flanking region	Augustus	3.3.3	*augustus*<br>--strand=forward \<br>--singlestrand=true \<br>--extrinsicCfgFile=extrinsic.cfg \<br>--alternatives-from-evidence=true \<br>--gff3=on \<br>--uniqueGeneId=true \<br>--UTR=off \<br>--species=fly \<br><input.fasta> > \<br><output.gff>	enoicyla_pusilla_h_fib.zip:<br>*augustus_h_fib_p_annotation.sh*<br>*augusuts_h_fib_hap2_annotation.sh*<br>*h2tg000016l_16866871_16911614_rev.fasta*<br>*h2tg000016l_16866871_16911614_rev.gff*<br>*ptg000034l_17642149_17687756.fasta*<br>*ptg000034l_17642149_17687756.gff*<br><br>NCBI GenBank: PX673985 and PX673986<br><br>philocasca_rivularis_h_fib.zip:<br>*augustus_h_fib_p_annotation.sh*<br>*augusuts_h_fib_hap2_annotation.sh*<br>*h2tg000051l_9731283_9778673.fasta*<br>*h2tg000051l_9731283_9778673.gz*<br>*ptg000016l_13013854_13061070_rev.fasta*<br>*ptg000016l_13013854_13061070_rev.gz*<br><br>NCBI GenBank: PX673987 and PX673988<br><br>supplementary notes S1 and S2, reported in main text, Table 2 and Table 3
identification of signal peptide	SignalP	6.0	https://services.healthtech.dtu.dk/services/SignalP-6.0/<br>organism = Eukarya, model mode = slow	supplementary notes S1 and S2
calculation of amino acid proportions	ExPASy ProtParam	n.a.	https://web.expasy.org/protparam<br>default	Appendix_S2_Amino_Acid_Composition_H_fibroin, reported in main text, Figure 4

Table S2: Blast hit table. Primary and alternate assemblies were blasted against termini of the h-fibroin of *Hesperophylax occidentalis* to identify the h-fibroin in the respective assemblies

**Query (*Hesperophylax occidentalis h-fibroin*)**	**Assembly**	**Hit contig**	**Hit start**	**Hit end**	**E-value**
MRAILLLIVFCSWQVHR (n-term)	JBPQUV000000000 (*E. pusilla)*	ptg000034l	17,652,149	17,652,199	1.80e-03
SVYTHPGINSVKSPCKLSDFNLLVKVGNVRKSNGNC (c-term)	JBPQUV000000000 (*E. pusilla)*	ptg000034l	17,677,646	17,677,756	2.89e-14
MRAILLLIVFCSWQVHR (n-term)	enoicyla_pusilla_alternate_assembly	h2tg000016l	16,901,614	16,901,564	1.78e-03
SVYTHPGINSVKSPCKLSDFNLLVKVGNVRKSNGNC (c-term)	enoicyla_pusilla_alternate_assembly	h2tg000016l	16,876,981	16,876,871	2.86e-14
MRAILLLIVFCSWQVHR (n-term)	JBPQUU000000000 (*P. rivularis)*	ptg000016l	13,051,070	13,051,020	8.13e-03
SVYTHPGINSVKSPCKLSDFNLLVKVGNVRKSNGNC (c-term)	JBPQUU000000000 (*P. rivularis)*	ptg000016l	13,023,964	13,023,854	1.72e-13
MRAILLLIVFCSWQVHR (n-term)	philocasca_rivularis_alternate_assembly	h2tg000051l	9,741,283	9,741,333	6.21e-03
SVYTHPGINSVKSPCKLSDFNLLVKVGNVRKSNGNC (c-term)	philocasca_rivularis_alternate_assembly	h2tg000051l	9,768,563	9,768,673	1.38e-13

Supplementary Figure S1: Genomescope Profile of *kmers* derived from HiFi reads (*Enoicyla pusilla*)

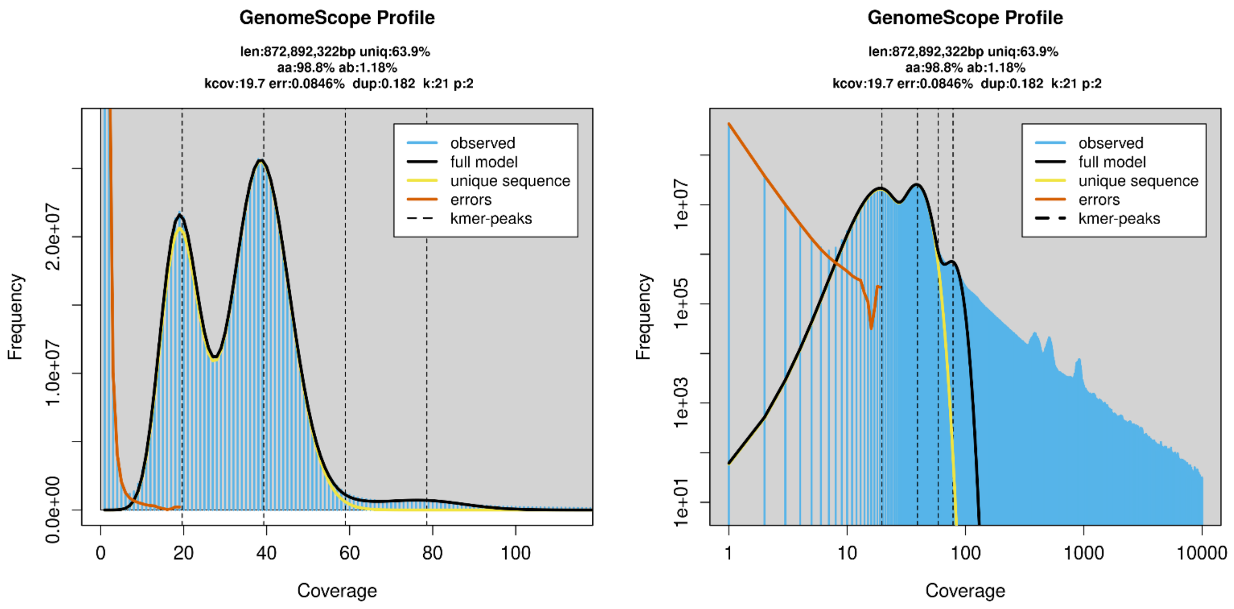

Supplementary Figure S2: Genomescope Profile of *kmers* derived from HiFi reads (*Philocasca rivularis)*

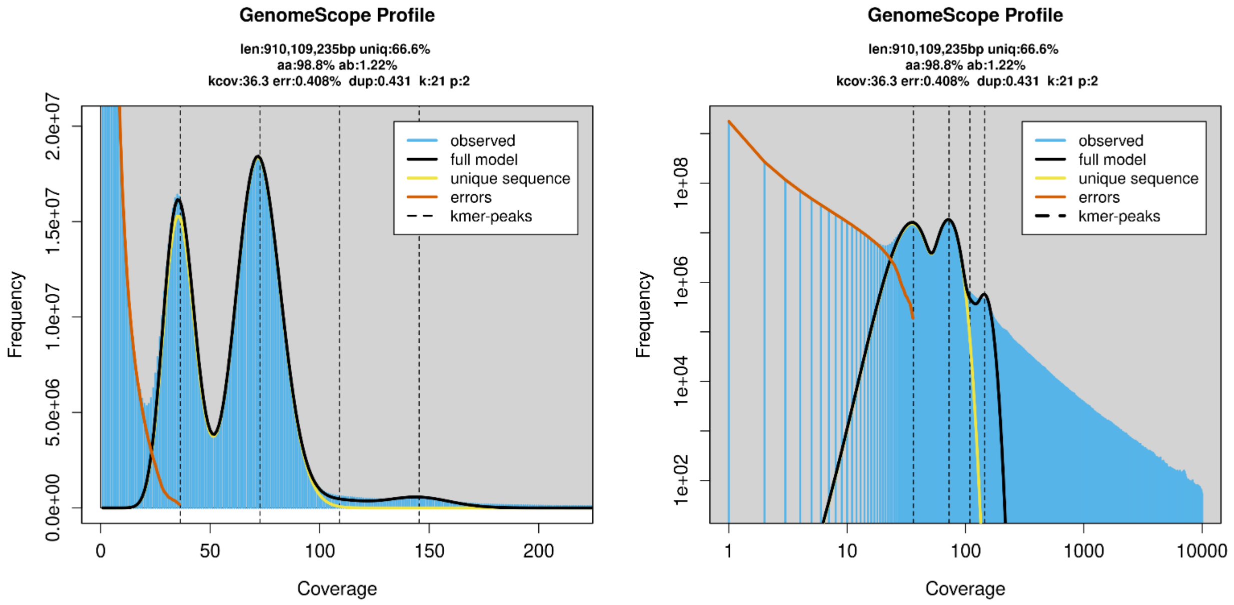

Supplementary Figure S3: Repeat landscape plot, where the x axis indicates divergence from consensus in Kimura distance, and the y axis indicates the percentage of the genome annotated as TE for each level of divergence. Ancient activity (greater divergence to consensus) appears on the left-hand side, while more recent activity is shown towards the right (greater similarity to consensus)

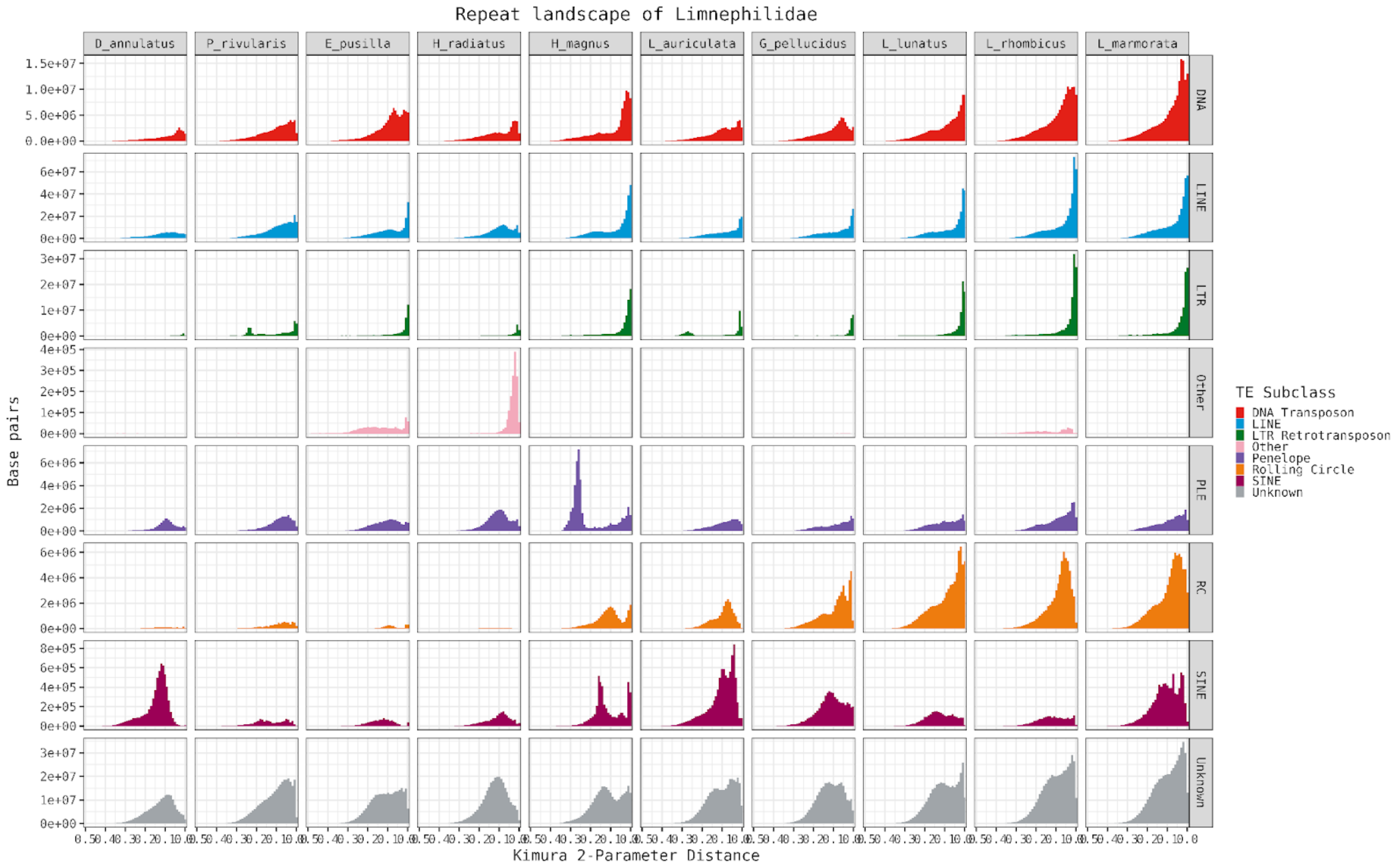

Supplementary Note S1: Schematic representation of the h-fibroin of *Enoicyla pusilla*

n-terminus (signal peptide)

MRAILLLIVFCSWQVQRTGSSAVSNKVQDLFRHGRHLDSDGLHERLLEEEDVIETNSKGEIIEKIISRKEIISDDTSNSWSESDSSESGSTEQIIKQIIIVQERPKHGHHHAKEKIYEEEIVIKKIGD

Transfer region

VVHGVDIPISIDGGLLRGGHGGRRGSKLSISRSVSIERIITPGVITKISRSSSVSIEVGRRGGLRGLGGLGGLSGSGDLVGLGGLGGLGGLRGLGGLRGLGGRRGKV

Repetitve central core

SISRSVSIERIVTPGSITKISRSSSVSIEVGRRGGLRGLGGLGGLSGSGDLVGLGGLGGLGGLRGLGGLRGLGGRRGKV (1)
SISRSVSIERIVTPGVITKISRSSSVSIEVGRRGGLRGLGGIGGLSGSGDLVGLGGLDGLGGLDGLGGLGGLDGLGGLGGLGGLRGLGGRRGYGL (2)
SESFSVSIERGIRRGPWGRRGKV (1)
SISRSVSIERIVTPGSITKISRSSSVSIEVGRRGGLGGLGGLRGLGGLRGLGGRRGKV (3)
SISRSVSIERIVTPGSITKISRSSSVSIEVGRRGGLRGLGGLGGLSGSGDLVGLGGLGGLGGLRGLGGLRGLGGRRGTV (4)
SISRSVSIERIVTPGSITKISRSSSVSIEVGRRGGLGGLGGLRGLGGLRGLGGRRGKV (5)
SISRSVSIERIVTPGSITKISRSSSVSIEVGRRGGLRGLGGLGGLSGSGDLVGLGGLDGLGGLDGLGGLGGLDGLGGLGGLGGLRGLGGRRGYGL (6)
SESFSVSIERGIRRGPWGRRGKV (2)
SISRSVSIERIVTPGVITKISRSSSVSIEVGRRGGLRGLGGLGGLSGSGDLVGLGGIDGLGGLDGLGGLGGLDGLGGLGGLGGLRGLGGRRGYGL (7)
SGSFSVSIERGIRRGPWGRRGKV (1)
SISRSVSIERIVTPGSITKISRSSSVSIEVGRRGGLRGLGGLGGLSGSGDLVGLGGLDGLGGLDGLGGLGGLDGLGGLGGLGGLRGLGGRRGYGL (8)
SGSFSVSIERGIRRGPWGRRGKV (2)
SISRSVSIERIVTPGSITKISRSSSVSIEVGRRGGLGGLGGLQGIGGLRGLGGRRGKV (9)
SISRSVSIERIVTPGSITKISRSSSVSIEVGRRGGLGGLGGLRGLGGLRGLGGRRGTV (10)
SISRSVSIERIVTPGVITKISRSSSVSIEVGRRGGLRGLGGLRGLSGSGDLVGLGGLDGLGGLDGLGDLGGLDGLGGLGGLGGLRGLGGRRGYGL (11)
SESFSVSIERGIRRGPWGRRGKV (3)
SISRSVSIERIVTPGVITKISRSSSVSIEVGRRGGLRGLGGLGGLSGSGDLVGLGGLDGLGGLDGLGGLGGLDGLGGLGGLGGLRGLGGRRGYGL (12)
SGSFSVSIERGIRRGPWGRRGKV (3)
SISRSVSIERIVTPGSITKISRSSSVSIEVGRRGGLGGLRGLVGLRGLGGRRGKV (13)
SISRSVSIERIVTPGSITKISRSSSVSIEVGRRGGLGGLGGLRGLGGLRGLGGRRGKV (14)
SISRSVSIERIVTPGVITKISRSSSVSIEVGRRGGLRGLGGLGVLSGSGDLVGLGGLDGLGGLDGLGGLGGLDGLGGLGGLGGLRGLGGRRGYGL (15)
SESFSVSIERGIRRGPWGRRGKV (4)
SISRSVSIERIVTPGVITKISRSSSVSIEVGRRGGLRGLGGLGGLSGSGDLVGLGGLDGLGGLDGLGGLGGLDGLGGLGGLGGLRGLGGRRGYGL (16)
SGSFSVSIERGIRRGPWGRRGKV (4)
SISRSVSIERIVTPGSITKISRSSSVSIEVGRRGGLGGLGGLRGLGGLRGLGGRRGKV (17)
SISRSVSIERIVTPGSITKISRSSSVSIEVGRRGGLGGLGGLRGLGGLRGLGGRRGKV (18)
SISRSVSIERIVTPGVITKISRSSSVSIEVGRRGGLRGLGGLGVLSGSGDLVGLGGLDGLGGLDGLGGLGGLDGLGGLGGLGGLRGLGGRRGYGL (19)
SGSFSVSIERGIRRGPWGRRGKV (5)
SISRSVSIERIVTPGSITKISRSSSVSIEVGRRGGLGGLGGLRGLGGLRGVGGRRGKV (20)
SISRSVSIERIVTPGVITKISRSSSVSIEVGRRGGLRGLGGLGGLSGSGDLVGLGGLDGLGGLDGLGGLGGLDGLGGLGGLGGLRGLGGRRGYGL (21)
SGSFSVSIERGIRRGPWGRRGKV (6)
SISRSVSIERIVTPGSITKISRSSSVSIEVGRRGGLGGLGGLRGLGGLRGLGGRRGKV (22)
SISRSVSIERIVTPGSITKISRSSSVSIEVGRRGGLGGLGGLRGLGGLRGLGGRRGKV (23)
SISRSVSIERIVTPGVITKISRSSSVSIEVGRRGGLRGLGGLGVLSGSGDLVGLGGLDGLGGLDGLGGLGGLDGLGGLGGLGGLRGLGGRRGYGL (24)
SGSFSVSIERGIRRGPWGRRGKV (7)
SISRSVSIERIVTPGSITKISRSSSVSIEVGRRGGLGGLGGLRGLGGLRGLGGRRGKV (25)
SISRSVSIERIVTPGSITKISRSSSVSIEVGRRGGLGGLGGLRGLGGLRGLGGRRGKV (26)
SISRSVSIERIVTPGVITKISRSSSVSIEVGRRGGLRGLGGLGVLSGSGDLVGLGGLDGLGGLDGLGGLGGLDGLGGLGGLGGLRGLGGRRGYGL (27)
SGSFSVSIERGIRRGPWGRRGKV (8)
SISRSVSIERIVTPGSITKISRSSSVSIEVGRRGGLGGLGGLRGLGGLRGLGGRRGKV (28)
SISRSVSIERIVTPGSITKISRSSSVSIEVGRRGGLGGLGGLGVLSGSGDLVGLGGLDGLGGLDGLGGLGGLDGLGGLGGLGGLRGLGGRRGYGL 29)
SESFSVSIERGIRRGPWGRRGKV (5)
SISRSVSIERIVTPGVITKISRSSSVSIEVGRRGGLRGLGGLGGLSGSGDLVGLGGLDGLGGLDGLGGLGRLDGLGGLGGLGGLRGLGGRRGYGL (30)
SGSFSVSIERGIRRGPWGRRGKV (9)
SISRSVSIERIVTPGSITKISRSSSVSIEVGRRGGLGGLGGLRGLGGLRGLGGRRGKV (31)

SISRSVSIERIVTPGSITKISRSSSVSIEVGRRGGLGGLGGLRGLGGLRGLGGRRGKV (32)
SISRSVSIERIVTPGVITKISRSSSVSIEVGRRGGLRGLGGLGVLSGSGDLVGLGGLDGLGGLDGLGGLGGLDGLGGLGGLGGLRGLGGRRGYGL (33)
SESFSVSIERGIRRGPWGRRGKV (6)
SISRSVSIERIVTPGVITKISRSSSVSIEVGRRGGLRGLGGLGGLSGSGDLVGLGGLDGLGGLDGLGGLGGLDGLGGLGGLGGLRGLGGRRGYGL (34)
SGSFSVSIERGIRRGPWGRRGKV (10)
SISRSVSIERIVTPGSITKISRSSSVSIEVGRRGGLGGLGGLRGLGGLRGLGGRRGKV (35)
SISRSVSIERIVTPGSITKISRSSSVSIEVGRRGGLGGLGGLRGLGGLRGLGGRRGKV (36)
SISRSVSIERIVTPGVITKISRSSSVSIEVGRRGGLRGLGGLGVLSGSGDLVGLGGLDGLGGLDGLGGLGGLDGLGGLGGLGGLRGLGGRRGYGL (37)
SESFSVSIERGIRRGPWGRRGKV (7)
SISRSVSIERIVTPGVITKISRSSSVSIEVGRRGGLRGLGGLGGLSGSGDLVGLGGLDGLGGLDGLGGLGGLDGLGGLGGLGGLRGLGGRRGYGL (38)
SGSFSVSIERGIRRGPWGRRGKV (11)
SISRSVSIERIVTPGSITKISRSSSVSIEVGRRGGLGGLGGLRGLGGLRGLGGRRGKV (39)
SISRSVSIERIVTPGSITKISRSSSVSIEVGRRGGLGGLGGLRGLGGLRGLGGRRGKV (40)
SISRSVSIERIVTPGVITKISRSSSVSIEVGRRGGLRGLGGLGVLSGSGDLVGLGGLDGLGGLDGLDGLGGLDGLGGLGGLDGLGGLGGLGGLRGLGGRRGYGL (41)
SESFSVSIERGIRRGPWGRRGKV (8)
SISRSVSIERIVTPGVITKISRSSSVSIEVGRRGGLRGLGGLGGLSGSGDLVGLGGLDGLGGLDGLGGLGGLDGLGGLGGLGGLRGLGGRRGYGL (42)
SGSFSVSIERGIRRGPWGRRGKV (12)
SISRSVSIERIVTPGSITKISRSSSVSIEVGRRGGLGGLGGLRGLGGLRGLGGRRGKV (43)
SISRSVSIERIVTPGSITKISRSSSVSIEVGRRGGLGGLGGLRGLGGLRGLGGRRGKV (44)
SISRSVSIERIVTPGVITKISRSSSVSIEVGRRGGLRGLGGLGVLSGSGDLVGLGGLDGLGGLDGLGGLGGLDGLGGLGGLGGLRGLGGRRGYGL (45)
SESFSVSIERGIRRGPWGRRGKV (9)
SISRSVSIERIVTPGVITKISRSSSVSIEVGRRGGLRGLGGLGGLSGSGDLVGLGGLDGLGGLDGLGGLGGLDGLGGLGGLGGLRGLGGRRGYGL (46)
SGSFSVSIERGIRRGPWGRRGKV (13)
SISRSVSIERIVTPGSITKISRSSSVSIEVGRRGGLGGLGGLRGLGGLRGLGGRRGKV (47)
SISRSVSIERIVTPGSITKISRSSSVSIEVGRRGGLGGLGGLRGLGGLRGLGGRRGKV (48)
SISRSVSIERIVTPGVITKISRSSSVSIEVGRRGGLRGLGGLGVLSGSGDLVGLGGLDGLGGLDGLDGLGGLDGLGGLGGLGGLRGLGGRRGYGL (49)
SESFSVSIERGIRRGPWGRRGKV (10)
SISRSVSIERIVTPGVITKISRSSSVSIEVGRRGGLRGLGGLGGLSGSGDLVGLGGLGGLGGLDGLGGLGGLDGLGGLGGLGGLRGLGGRRGYGL (50)
SESFSVSIERGIRRGPWGRRGKV (11)
SISRSVSIERIVTPGVITKISRSSSVSIEVGRRGGLRGLGGLGGLSGSGDLVGLGGLDGLGGLDGLGGLGGLDGLGGLGGLGGLRGLGGRRGYGL (51)
SGSFSVSIERGIRRGPWGRRGKV (14)
SISRSVSIERIVTPGSITKISRSSSVSIEVGRRGGLGGLGGLRGLGGLRGLGGRRGKV (52)
SISRSVSIERIVTPGSITKISRSSSVSIEVGRRGGLGGLGGLRGLGGLRGLGGRRGKV (53)
SISRSVSIERIVTPGVITKISRSSSVSIEVGRRGGLRGLGGLGVLSGSGDLVGLGGLDGLGGLDGLGGLGGLDGLGGLGGLGGLRGLGGRRGYGL (54)
SESFSVSIERGIRRGPWGRRGKV (12)
SISRSVSIERIVTPGVITKISRSSSVSIEVGRRGGLRGLGGLGGLSGSGDLVGLGGLDGLGGLDGLGGLGGLDGLGGLGGLGGLRGLGGRRGYGL (55)
SGSFSVSIERGIRRGPWGRRGKV (15)
SISRSVSIERIVTPGSITKISRSSSVSIEVGRRGGLGGLGGLRGLGGLRGLGGRRGKV (56)
SISRSVSIERIVTPGSITKISRSSSVSIEVGRRGGLGGLGGLRGLGGLRGLGGRRGKV (57)
SISRSVSIERIVTPGSITKISRSSSVSIEVGRRGGLGGLGGLRGLGGLRGLGGRRGKV (58)
SISRSVSIERIVTPGVITKISRSSSVSIEVGRRGGLRGLGGLGVLSGSGDLVGLGGLDGLGGLDGLGGLGGLDGLGGLGGLGGLRGLGGRRGYGL (59)
SGSFSVSIERGIRRGPWGRRGKV (16)
SISRSVSIERIVTPGSITKISRSSSVSIEVGRRGGLGGLGGLRGLGGLRGLGGRRGKV (60)
SISRSVSIERIVTPGSITKISRSSSVSIEVGRRGGLGGLGGLRGLGGLRGLGGRRGKV (61)
SISRSVSIERIVTPGVITKISRSSSVSIEVGRRGGLRGLGGLGVLSGSGDLVGLGGLDGLGGLDGLGGLGGLDGLGGLGGLGGLRGLGGRRGYGL (62)
SGSFSVSIERGIRRGPWGRRGKV (17)
SISRSVSIERIVTPGSITKISRSSSVSIEVGRRGGLGGLGGLRGLGGLRGLGGRRGKV (63)
SISRSVSIERIVTPGSITKISRSSSVSIEVGRRGGLGGLGGLRGLGGLRGLGGRRGKV (64)
SISRSVSIERIVTPGVITKISRSSSVSIEVGRRGGLRGLGGLGVLSGSGDLVGLGGLDGLGGLDGLDGLGGLDGLGGLGGLGGLRGLGGRRGYGL (65)
SESFSVSIERGIRRGPWGRRGKV (13)
SISRSVSIERIVTPGVITKISRSSSVSIEVGRRGGLRGLGGLGGLSGSGDLVGLGGLDGLGGLDGLGGLGGLDGLGGLGGLGGLRGLGGRRGYGL (66)
SGSFSVSIERGIRRGPWERRGKV (18)

SISRSVSIERIVTPGSITKISRSSSVSIEVGRRGGLGGLGGLRGLGGLRGLGGRRGKV (67)
SISRSVSIERIVTPGSITKISRSSSVSIEVGRRGGLGGLGGLRGLGGLRGLGGRRGKV (68)
SISRSVSIERIVTPGVITKISRSSSVSIEVGRRGGLRGLGGLGVLSGSGDLVGLGGLDGLGGLDGLGGLGELDGLGGLGGLGGLRGLGGRRGYGL (69)
SESFSVSIERGIRRGPWGRRGKV (14)
SISRSVSIERIVTPGVITKISRSSSVSIEVGRRGGLRGLGGLGGLSGSGDLVGLGGLDGLGGLDGLGGLGGLDGLGGLGGLGGLRGLGGRRGYGL (70)
SGSFSVSIERGIRRGPWGRRGKV (19)
SISRSVSIERIVTPGSITKISRSSSVSIEVGRRGGLGGLGGLRGLGGLRGLGGRRGKV (71)
SISRSVSIERIVTPGSITKISRSSSVSIEVGRRGGLGGLGGLRGLGGLRGLGGRRGKV (72)
SISRSVSIERIVTPGSITKISRSSSVSIEVGRRGGLGGLGGLRGLGGLRGLGGRRGKA (73)
SISRSVSIERIVTPGVITKISRSSSVSIEVGRRGGLRGLGGLGVLSGSGDLVGLGGLDGLGGLDGLDGLGGLDGLGGLGGLGGLRGLGGRRGYGL (74)
SESFSVSIERGIRRGPWGRRGKV (15)
SISRSVSIERIVTPGVITKISRSSSVSIEVGRRGGLRGLGGLGGLSGSGDLVGLGGLDGLGGLDGLGGLGGLDGLGGLGGLGGLRGLGGRRGYGL (75)
SGSFSVSIERGIRRGPWGRRGKV (20)
SISRSVSIERIVTPGSITKISRSSSVSIEVGRRGGLGGLGGLRGLGGLRGLGGRRGKV (76)
SISRSVSIERIVTPGSITKISRSSSVSIEVGRRGGLGGLGGLRGLGGLRGLGGRRGKV (77)
SISRSVSIERIVTPGVITKISRSSSVSIEVGRRGGLRGLGGLGVLSGSGDLVGLGGLDGLGGLDGLDGLGGLDGLGGLGGLGGLRGLGGRRGYGL (78)
SESFSVSIERGIRRGPWGRRGKV (16)
SISRSVSIERIVTPGVITKISRSSSVSIEVGRRGGLRGLGGLGGLSGSGDLVGLGGLDGLGGLDGLGGLGGLDGLGGLGGLGGLRGLGGRRGYGL (79)
SGSFSVSIERGIRRGPWGRRGKV (21)
SISRSVSIERIVTPGSITKISRSSSVSIEVGRRGGLGGLGGLRGLGGLRGLGGRRGKV (80)
SISRSVSIERIVTPGSITKISRSSSVSIEVGRRGGLGGLGGLRGLGGLRGLGGRRGKV (81)
SISRSVSIERIVTPGVITKISRSSSVSIEVGRRGGLRGLGGLGVLSGSGDLVGLGGLDGLGGLDGLDGLGGLDGLGGLGGLGGLRGLGGRRGYGL (82)
SESFSVSIERGIRRGPWGRRGKV (17)
SISRSVSIERIVTPGVITKISRSSSVSIEVGRRGGLRGLGGLGGLSGSGDLVGLGGLDGLGGLDGLGGLGGLDGLGGLGGLGGLRGLGGRRGYGL (83)
SGSFSVSIERGIRRGPWERRGKV (22)
SISRSVSIERIVTPGSITKISRSSSVSIEVGRRGGLGGLGGLRGLGGLRGLGGRRGKV (84)
SISRSVSIERIVTPGSITKISRSSSVSIEVGRRGGLGGLGGLRGLGGLRGLGGRRGKV (85)
SISRSVSIERIVTPGVITKISRSSSVSIEVGRRGGLRGLGGLGVLSGSGDLVGLGGLDGLGGLDGLGGLGGLDGLGGLGGLGGLRGLGGRRGYGL (86)
SESFSVSIERGIRRGPWGRRGKV (18)
SISRSVSIERIVTPGVITKISRSSSVSIEVGRRGGLRGLGGLGGLSGSGDLVGLGGLDGLGGLDGLGGLGGLDGLGGLGGLGGLRGLGGRRGYGL (87)
SGSFSVSIERGIRRGPWGRRGKV (23)
SISRSVSIERIVTPGSITKISRSSSVSIEVGRRGGLGGLGGLRGLGGLRGLGGRRGKV (88)
SISRSVSIERIVTPGSITKISRSSSVSIEEGRRGGLRGLGGLGGLSGSGDLDVLGGLGGLGGLRGLGGLRGLGGRRGKV (89)
SISRSVSIERIVTPGSITKISRSSSVSIEVGRRGGLGGLGGLRGLGGLRGLGGRRGKV (90)

Transfer region

SISRSVSFERIVTPGSITKISRSSSVSIEVGRRGGLGGLGGLRGLGGLRGLGGRRGKVSISRSVSIERIVTPGVITEISRQSSVSIEVGRRGGLRGLGGLVGLGGRRRKVSVSQSVSIERIVSPGVITKISRSSSVSIEVGRRGGLRGLGGLGGLGGRRRQVSISQS
VSIERIVTPGVITKISRSSSISIEGGRRRGPWGYGRGLAGLSGSENLDELGGLGGLVALGRPGVV

c-terminus

PVPSVYTNHPGISSVKSPCKLSDFNLLVKVGNVRKTIGNC*

Supplementary Note S2: Schematic representation of the h-fibroin of *Philocasca rivularis*

n-terminus (signal peptide)

MRAILLLIVFCSWQVHRTGSSAVSNKVQDLFRHGRHLDSDGLHERLLEEEDVIETNSKGEIIEKIISRKEIISDDASDSWSDSDSSESGSTEQIIKQIIIVQEKPKHGHHHAKEKIYEEEIVIKKIGD

Transfer region

VVHGVNYPTGYLSNDEGLRLEGRGGRRGSKLSISRSVSIEQVIRPSVYTKISKSSSVSIEGGRGRGPWGYGRGAWGSGDLDELGGLGGLGGYGRGI

Repetitive central core

SGSLSVSVERSYRRGPWGRRGKVSISRSVSIERIVTPGVYTKISRSSSVSVEGGLRRGPWGYGRELGGYGVGI (1)
SGSVSVSIERGIRRGPWGRRGKVSISRSVSIERIVTPGVYTHISRSSSVSVEGGRRRGPWGYGRGLGGYGVGI (1)
SGSLSVSVERSYRRGPWGRRGKVSISRSVSIERIVTPGVYTHISRSSSVSVEGGRRGGLWGYGQGLGRNGVGI (2)
SGSLSVSVERSYRRGPWGRRGKVSISRSVSIERIVTPGVYTKISRSSSVSVEGGRRRGPWGYGRGLGGLSGSGDLDGLGGYGGLDGYGSLGGLGGYGGLGGYGGI (2)
SGSLSVSVERSYRRGPWGRRGKVSISRSVSIERIVTPGVYTKISRSSSVSVEGGRRGGLWGYGRGLGGLSGSGDLDGLDGLGGYGRGPWGYGRGLGGLGGSGDRDGLDGLGGLGGYGHGYGLGLGGLGGSGDLDGLGGYGGLGGYGGLGGLGGY
GGLRGYGGGI (3)
SGSLSVSVERGYRRGPWGRRGKVSISRSVSIERIVTPGVYTHISRSSSVSVEGGRRRGPWGYGRGLGGLSGSGDLDGLGGYGGLDGYGGLGGLGGYGGLGGYGGI (4)
SGSLSVSVERSYRRGPWGRRGKVSISRSVSIERIVTPGVYTKISRSSSVSVEGGRRRGPWGYGRGLGGLSGSGDLDGLGGYGGLDGYGGLGGLGGYGGLGGYGGI (5)
SGSLSVSVERSYRRGPWGRRGKVSISRSVSIERIVTPGVYTKISRSSSVSVEGGRRGGLWGYGRGLGGLSGSGDLDGLDGLGGLGGYGHGYGLGLGGLGGSGDLDGLGGYGGLGGYGGLGGLGSYGGLGGYGGGI (6)
SGSLSVSVERGYGRGPWGRRGKVSISRSVSIERIVTPGVYTHISRSSSVSVEGGRRRGPWGYGRGLGGLSGSGDLDGLGGYGGLGGLGGYGGLGGYGGGI (7)
SGSLSVSVERGYRRGPWGRRGKVSISRSVSIERIVTPGLYTKISRSSSVSIEGGRRRGPWGYGRGLGGLSGSGDLDGLDGLGGLGGYGHGYGLGLGGLGGSGDLDGLGGYGGLGGYGGLGGLGGYGGLGGYGGGI (1)
SGSLSVSVERGYGRRPWGRRGKVSISRSVSIERIVTPGVYTHISRSSSVSVEGGRRRGPWGYGRGLGGLSGSGDLDGLDGLGGLGGYGHGYGLGLGGLGGSGDLDGLGGYGGLGGYGGLGGLGGYGGLGGYGGGI (8)
SGSLSVSVERGYRRGPWGRRGKVSISRSVSIERIVTPGVYTHISRSSSVSVEGGRRRGPWGYGRGLGGLSGSGDLDGLGGYGGLGGLGGYGGLGGYGGGI (9)
SGSLSVSVERGYRRGPWGRRGKVSISRSVSIERIVTPGVYTKISRSSSVSIEGGRRRGPWGYGRGLGGLSGSGDLDGLDGLGGLGGYGHGYGLGLGGLGGSGDLDGLGGYGGLGGYGGLDGYGGLGGLGGYGGLGGYGGI (2)
SGSLSVSVERSYRRGPWGRRGKVSISRSVSIERIVTPGVYTKISRSSSVSIEGGRRRGPWGYGRGLGGLSGSGDLDGLDGLGGLGGYGHGYGLGLGGLGGSGELDGLGGYGGLGGYGGLGGLGGYGGLGGYGGGI (3)
SGSLSVSVERGYRRGPWGRRGKVSISRSVSIERIVTPGVYTHISRSSSVSVEGGRRRGPWGYGRGLGGLSGSGDLDGLGGYGGLGGLGGYGGLGGYGGGI (10)
SGSLSVSVERGYRRGPWGRRGKVSISRSLSIERIVTPGVYTHISRSSSVSVEGGRRRGPWGYGRGLGGLSGSGDLDGLGGYGGLDGYGGLGGLGGYGGLGGYGGI (1)
SGSLSVSVERSYRRGPWGRRGKVSISRSVSIERIVTPGVYTKISRSSSVSIEGGRRRGPWGYGRGLGGLSGSGDLDGLDGLGGLGGYGHGYGLGLGGLGGSGDLDGLGGYGGLGGYGGLGGYGGLDGYGGLGGLGGYGGLGGYGGI (4)
SGSLSVSVERSYRRGPWGRRGKVSISRSVSIERIVTPGVYTKISRSSSVSIEGGRRRGPWGYGRGLGGLSGSGDLDGLDGLGGLGGYGHGYGLGLGGLGGSGDLDGLGGYGGLGGYGGLGGLGGYGGLGGYGGGI (5)
SGSLSVSVERGYRRGPWGRRGKVSISRSVSIERIVTPGVYTHISRSSSVSVEGGRRRGPWGYGRGLGGLSGSGDLDGLGGYGGLGGLGGYGGLGGYGGGI (11)
SGSLSVSVERGYRRGPWGRRGKVSISRSLSIERIVTPGVYTHISRSSSVSVEGGRRRGPWGYGRGLGGLSGSGDLDGLGGYGGLDGYGGLGGLGGYGGLGGYGGV (2)
SGSLSVSVERSYRRGPWGRRGKVSISRSVSIERIVTPGVYTKISRSSSVSIEGGRRRGPWGYGRGLGGLSGSGDLDGLDGLGGLGGYGHGYGLGLGGLGGSGDLDGLGGYGGLGGYGGLDGYGGLGGYGGI (6)
SGSLSVSVERSYRRGPWGRRGKVSISRSVSIERIVTPGVYTKISRSSSVSIEGGRRRGPWGYGRGLGGLSGSGDLDGLDGLGGLGGYGHGYGLGLGGLGGSGDLDGLGGYGGLGGYGGLGGLGGYGGLGGYGGGI (7)
SGSLSVSVERGYRRGPWGRRGKVSISRSVSIERIVTPGVYTHISRSSSVSVEGGRRRGPWGYGRGLGGLSGSGDLDGLGGYGGLGGLGGYGGLGGYGGGI (12)
SGSLSVSVERGYRRGPWGRRGKVSISRSLSIERIVTPGVYTHISRSSSVSVEGGRRRGPWGYGRGLGGLSGSGDLDGLGGYGGLDGYGGLGGLGGYGGLGGYGGI (3)
SGSLSVSVERSYRRGPWGRRGKVSISRSVSIERIVTPGVYTKISRSSSVSIEGGRRRGPWGYGRGLGGLSGSGDLDGLDGLGGLGGYGHGYGLGLGGLGGSGDLDGLGGYGGLGGYGGLGGLGGYGGLGGYGGGI (8)
SGSLSVSVERGYRRGPWGRRGKVSISRSVSIERIVTPGVYTHISRSSSVSVEGGRRRGPWGYGRGLGGLSGSGDLDGLGGYGGLGGLGGYGGLGGYGGGI (13)
SGSLSVSVERGYRRGPWGRRGKVSISRSLSIERIVTPGVYTHISRSSSVSVEGGRRRGPWGYGRGLGGLSGSGDLDGLGGYGGLDGYGGLGGLGGYGGLGGYGGI (4)
SGSLSVSVERSYRRGPWGRRGKVSISRSVSIERIVTPGVYTKISRSSSVSIEGGRRRGPWGYGRGLGGLSGSGDLDGLDGLGGLGGYGHGYGLGLGGLGGSGDLDGLGGYGGLGGYGGLDGYGGLGGLGGYGGLGGYGGI (9)
SGSLSVSVERSYRRGPWGRRGKVSISRSVSIERIVTPGVYTKISRSSSVSIEGGRRRGPWGYGRGLGGLSGSGDLDGLDGLGGLGGYGHGYGLGLGGLGGSGDLDGLGGYGGLGGYGGLGGLGGYGGLGGYGGGI (10)
SGSLSVSVERGYRRGPWGRRGKVSISRSVSIERIVTPGVYTHISRSSSVSVEGGRRRGPWGYGRGLGGLSGSGDLDGLGGYGGLGGLGGYGGLGGYGGGI (14)
SGSLSVSVERGYRRGPWGRRGKVSISRSLSIERIVTPGVYTHISRSSSVSVEGGRRRGPWGYGRGLGGLSGSGDLDGLGGYGGLDGYGGLGGLGGYGGLGGYGGI (5)
SGSLSVSVERSYRRGPWGRRGKVSISRSVSIERIVTPGVYTKISRSSSVSIEGGRRRGPWGYGRGLGGLSGSGDLDGLDGLGGLGGYGHGYGLGLGGLGGSGDLDGLGGYGGLGGYGGLDGYGGLGGLGGYGGLGGYGGI (11)
SGSLSVSVERSYRRGPWGRRGKVSISRSVSIERIVTPGVYTKISRSSSVSIEGGRRRGPWGYGRGLGGLSGSGDLDGLDGLGGLGGYGHGYGLGLGGLGGSGDLDGLGGYGGLGGYGGLGGLGGYGGLGGYGGGI (12)
SGSLSVSVERGYRRGPWGRRGKVSISRSVSIERIVTPGVYTHISRSSSVSVEGGRRRGPWGYGRGLGGLSGSGDLDGLGGYGGLDGYGGLGGLGGYGGLGGYGGI (15)
SGSLSVSVERGYRGGPWGRRGKVSISRSVSIERIVTPGVYTHISRSSSVSVEGGRRGGLWGYGRGLGGLSGSGDLDGLGGYGGLGGLGGYGGLGGYGGLGGYGGI (16)
SGSLSVSVERSYGRGPWGRRGKVSISRSVSIERIVSPGVYTHISRSSSVSVEGGLRRGPWGYGRGLGGYGVGI (17)
SGSLSVSVERSYRRGPWGRRGKVSISRSVSVERIVTPGVYTHISRSSSVSVEGGRRGGLWGYGRGLGGLSGSGDLDGLGGYGGLGGLGGYGGLGGYGGI (1)
SGSLSVSVERSYRRGPWGRRGKVSISRSVSIERIVTPGVYTHISRSSSVSVEGGRRGGLWGYGRGLGGLSGSGDLDGLDGLGGYGRGPWGYGRGLGGLSGSGDLDGLGGYGGLDGYGGLGGLGGYGGLGGLGGYDGLGGLGSYGGLGGYGGI
(18)
SGSLSVSVERGYRRGPWGRRGKVSISRSVSIERIVTPGVYTKISRSSSVSIEGGRRRGPWGYGRGLGGLSGSGDLDGLDGLGGLGGYGHGYGLGLGGLGGSGDLDGLGGYGGLGGYGGLGGLGGYGGLGGYGGGI (13)
SGSLSVSVERSYRRGPWGRRGKVSISRSVSIERIVTPGVYTKISRSSSVSIEGGRRRGPWGYGRGLGGLSGSGDLDGLGGYGGLDGYGGLGGLGGYGGLGGLGGYDGLGGLGGYGGLGGYGGI (14)
SGSLSVSVERGYRRGPWGRRGKVSISRSVSIERIVTPGVYTHISRSSSVSVEGGRRGGLWGYGRGLGGLSGSGDLDGLGGYGGLGGLGGYGGLGGYGGLGGYGGI (19)
SGSLSVSVERSYGRGPWGRRGKVSISRSVSIERIVTPGVYTHISRSSSVSVEGGLRRGPWGYGRGLGGYGVGI (20)
SGSLSVSVERSYRRGPWGRRGKVSISRSVSIERIVTPGVYTHISRSSSVSVEGGRRGGLWGYGRGRGGLSGSGDLDGLGGYGGLDGYGGLGGVGGYGGLGGLGGYGGLGGYGGI (21)

SGSLSVSVERSYRRGPWGRRGKVSISRSVSIERIVTPGVYTKISRSSSVSVEGGRRGGLWGYGRGLGGLSGSGDLDGLDGLGGYGRGPWGYGRGLGGLGGSGDLDGLGGYGGLDGYGGLGGLGGYGGLGGYGGI (22)
SGSLSVSVERGYRRGPWGRRGKVSISRSVSIERIVTPGVYTHISRSSSVSVEGGRRGGPWGYGRGLGGLSGSGDLDGLDGLGGYGRGPWGYGRGLGGLSGSGDLDGLGGYGGLDGYGGLGGLGGYGGLGGLGGYGGLGGYGGI (23)
SGSLSVSVERGYRRGPWGRRGKVSISRSVSIERIVTPGVYTKISRSSSVSVEGGRRGGLWGYGRGLGGLSGSGDLDGLGGYGGLDGYGGLGGLGGYGGLGGLGGYGGLGGYGGI (24)
SGSLSVSVERGYRRGPWGRRGKVSISRSVSIERIVTPGVYTKISRSSSVSIEGGRRGGLWGYGRGLGGLSGSGDLDGLGGYGGLDGYGGLGGLGGYGGLGGLGGYGGPGGYGGI (15)
SGSLSVSVERSYRRGPWGRRGKVSISRSVSIERIVTPGVYTKISRSSSVSVEGGRRGGLWGYGRGLGGLSGSGDLDGLDGLGGYGRGPWGYGRGLGGLGGSGDLDGLDGRGGLGGYGHGYGLGLGGLGGSGDLDGLGGYGGLDGYGGLGGLGGY
GGLGGYGGI (25)
SGSLSVSVERGYRGGPWGRRGKVSISRSVSIERIVTPGVYTHISRSSSVSVEGGRRGGLWGYGRGLGGLSGSGDLDGLGGYGGLGGLGGYGGLGGYGGLGGYGGI (26)
SGSLSVSVERSYGRGPWGRRGKVSISRSLSIERIVSPGVYTHISRSSSVSVEGGRLGGPWGYGRGLGGYGVGI (6)
SGSLSVSVERSYRRGPWGRRGKVSISRSVSIERIVTPGVYTHISRSSSVSVEGGLRRGPWGYGRGLGGYGVGI (27)
SGSLSVSVERSYRRGPWGRRGKVSISRSVSVERIVTPGVYTHISRSSSVSVEGGRRGGLWGYGRGLGGLSGSGDLDGLDGLGGYGRGPWGYGRGLGGLSGSGDLDGLGGYGGLGGLGGYGGLGGLGGYDGLGGLVGYGGLGGYGGI (2)
SGSLSVSVERGYRRGPWGRRGKVSISRSVSIERIVTPGVYTHISRSSSVSVEGGRRGGLWGYGRGLGGLSGSGDLDGLDGLGGYGRGPWGYGRGLGGLSGSGDLDGLGGYGGLDGYGGLGGLGGYGGLGGLGGYDGLGGLGGYGGLGGYGGI
(28)
SGSLSVSVERGYRRGPWGRRGKVSISRSVSIERIVTPGVYTHVSRSSSVSVEGGRRGGLWGYGRGLGGLSGSGDLDGLGGYGGLGGLGGYGGLGGYGGI (29)
SGSLSVSVERSYRRGPWGRRGKVSISRSVSIERIVTPGVYTHISRSSSVSVEGGRRGGLWGYGRGLGGLSGSGDLDGLDGLGGYGRGPWGYGRGLEGLSGSGDLDGLGGYGGLDGYGGLGGLGGYGGLGGLGGYDGLGGLGGYGGLGGYGGI
(30)
SGSLSVSVERGYRRGPWGRRGKVSISRSVSIERIVTPGVYTHISRSSSVSVEGGRRGGLWGYGRGLGGLSGSGDLDGLDGLGGYGRGPWGYGRGLGGLSGSGDLDGLGGYGGLDGYGGLGGLGGYDGLGGLGGYGGLGGYGGI (31)
SGSLSVSVERGYRRGPWGRRGKVSISRSVSIERIVTPGVYAHISRSSSVSVEGGRRGGLWGYGRGLEGLSGSGDLDGLGAYGGLGGLGGYGGLGGYGGLGGYGGI (32)
SGSLSVSVERSYGRGPWGRRGKVSISRSVSIERIVTPGVYTHISRSSSVSVEGGLRRGPWGYGRGLGGYGVGI (33)
SGSLSVSVERSYRRGPWGRRGKVSISRSVSIERIVTPGVYTHISRSSSVSVEGGRRGGLWGYGRGLGGLSGSGDLDGLGGYGGLGGLGSYGGLGGYGGI (34)
SGSLSVSVERSYRRGPWGRRGKVSISRSVSIERIVTPGVYTHISRSSSVSVEGGRRGGLWGYGRGLGGLSGSGDLDGLDGLGGYGRGPWGYGRGLGGLSGSGDLDGLGGYGGLDGYGGLGGLGGYGSLGGLGGYGGLGGYGGI (35)
SGSLSVSVERSYRRGPWGRRGKVSISRSVSIERIVTPGVYTHISRSSSVSVEGGRRGGLWGYGRGLGGLSGSGDLDGLDGLGGYGRGPWGYGRGLGGLSGSGDLDGLGGYGGLDGYGGLGGLGGYGGLGGYGGI (36)
SGSLSVSVERGYRRGPWGRRGKVSISRSVSIERIVTPGVYTHISRSSSVSVEGGRRGGLWGYGRGLGGLSGSGDLDGLDGLGGYGRGPWGYGRGLGGLSGSGDLDGLGGYGGLDGYGGLGGLGGYGGLGGLGGYGGLGGYGGI (37)
SGSLSVSVERGYRRGPWGRRGKVSISRSVSIERIVTPGVYTKISRSSSVSIEGGRRGGVWGYGRGLGGLSGSGDLDGLDGLGGYGRGPWGYGRGLGGLSGSGDLDGLGGYGGLDGYGGLGGLGGYGGLGGLGGYDGLGGLGGYGGLGGYGGI
(16)
SGSLSVSVERGYRRGPWGRRGKVSISRSVSIERIVTPGVYAHISRSSSVSVEGGRRGGLWGYGRGLEGLSGSGDLDGLGGYGGYGGLGGYGGLGGYGGLGGYGGI (38)
SGSLSVSVERSYGRGPWGRRGKVSISRSVSIERIVTPGVYTHISRSSSVSVEGGLRRGPWGYGRGLGGYGVGI (39)
SGSLSVSVERSYRRGPWGRRGKVSISRSVSIERIVTPGVYSHISRSSSVSVEGGRRGGLWGYGRGLGGLSGSGDLNGLGGYGGLGGLGGYGGLGGYGGI (40)
SGSLSVSVERSYRRGPWGRRGKVSISRSVSIERIVTPGVYTHISRSSSVSVEGRRRGGLWGYGRGLGGLSGSGDLDGLDGLGGYGRGPWGYGRGLGGLSGSGDLDGLGGYGGLDGYGGLGGLGGYGGLGGLGGYGGLGGYGGI (42)
SGSLSVSVERGYRRGPWGRRGKVSISRSVSIERIVTPGVYTKISRSSSVSVEGGRRGGLWGYGRGLGGLSGSGDLDGLGGYGGLDGYGGLGGLGGYGGLGGLGGYGGLGGYGGI (42)
SGSLSVSVERGYRRGPWGRRGKVSISRSVSIERIVTPGVYTKISRSSSVSVEGGRRGGLWGYGRGLGGLSGSGDLDGLDGLGGYGRGPWGYGRGLGGLSGSGDLDGLGGYGDLDGYGGLGGLGGYGGLGGLGGYGGLGGYGGI (43)
SGSLSVSVERGYRRGPWGRRGKVSISRSVSIERIVTPGVYTKISRSSSVSVEGGRRAGLWGYGRGLGGLSGSGDLDGLDGLGGYGRGPWGYGRGLGGLSGSGDLDGLGGYGGLDGYGGLGGLGGYGGLGGLGGYGGLGGYEGI (44)
SGSLSVSVERGYRRGPWGRRGKVSISRSVSIERIVTPGVYTHISRSSSVSVEGGRRGGLWGYGRGLEGLSGSGDLDGLGGYGGLGGLGGYGGLGGYGGLGGYGGI (45)
SGSLSVSVERSYGRGPWGRRGKVSISRSVSIERIVTPGVYTHISRSSSVSVEGGRRGGPWGYGRGLGGYGVGI (46)
SGSLSVSVERSYRRGPWGRRGKVSISRSVSIERIVTPGVYTHISRSSSVSVESGRRRGIWAGRLGKV (47)

Transfer region

SISRSVSVERVIRPLVYSNISGSSSISVERGIRRGPLGRLGGYGLSGSSSIESLRPGRVLAGLGRVGALGRSGVV

c-terminus

PVPSVYTNHPGLKSVKAPCKLSDYNLLVKVGSVRKSNGNC*
